# The Body Politic: Leftist Humanitarianism in Latin American Social Medicine

**DOI:** 10.1177/00220094251401605

**Published:** 2025-12-12

**Authors:** Sebastian Fonseca

**Affiliations:** History and Archeology, 3286 University of Exeter, Exeter, UK

**Keywords:** Cold War, grassroots movement, Latin American left, leftist humanitarianism, social medicine, transnational networks

## Abstract

This article examines the transnational network of Latin American Social Medicine, represented by the Latin American Social Medicine Association (ALAMES), as a distinctive form of leftist humanitarianism that emerged during the Cold War. Amid fierce state-sponsored campaigns of anti-communist terror aimed at leftist ‘epistemicide’, this intellectual community was targeted for its critical, politically active stance on health. The article argues that the very effort to destroy the network paradoxically spurred its transformation into a resilient South-South solidarity movement. Drawing on oral histories and archival research, it distinguishes ALAMES's humanitarianism from both traditional, impartial aid and the post-1970s ‘new humanitarianism’ based on liberal human rights discourse and NGOs. While other solidarity movements employed legalistic strategies, ALAMES's humanitarian approach concentrated on creating and maintaining a critical epistemology rooted in historical materialism. It functioned by protecting persecuted practitioners, establishing institutional safe havens in Mexico and Brazil, and circulating clandestine texts (the ‘*Libro Gris’*). This history presents a decolonial counter-narrative to Euro-US accounts of aid, demonstrating how solidarity founded on a shared struggle dissolved the donor-recipient binary to generate a powerful critique of modern technocratic global health. It offers a framework for epistemic survival in contemporary conflicts where knowledge producers are deliberately targeted.

August 1987 was meant to be a moment of political triumph for Latin American social medicine in Medellin, Colombia. A few years before, the newly established Latin American Social Medicine Association (ALAMES) agreed to host its inaugural conference at the *Universidad de Antioquia*, presided over by the elected physician director, Saul Franco.^1^ But what began as a beacon of hope for health equity quickly descended into a coordinated campaign of terror aligned with the most violent version of the country's anti-communism. For Franco and colleagues, the event became an epicentre of an intellectual purge, a violent crusade against ideas, and the epistemicide of leftist movements.^2^

Amidst fierce political backlash against the socialist party ‘*Union Patriotica’* (UP), paramilitary death squads, acting in concert with state forces, began to hunt the city's most prominent leftist leaders.^3^ Their mission, in the chilling words of commander Carlos Castaño, was to ‘*anular cerebros*’ – to cancel out brains and systematically extinguish the intellectual sources of social justice.^4^ Esteemed physicians like Hector Abad-Gomez, Leonardo Betancourt, and UP Senator Pedro Luis Valencia were assassinated, their deaths intended to shatter a movement (Figure 1).^5^ Caught in this maelstrom, Franco saw his friends buried and soon discovered his own name on a death list. Yet, in the face of annihilation, a lifeline emerged. Relying on contacts within the social medicine network, Franco obtained funding for a doctoral degree at the *Fundação Oswaldo Cruz* (*Fiocruz*, Rio de Janeiro, Brazil). Within hours of Abad-Gomez's murder, Franco was boarding a plane to start over in a foreign country – exiled not by chance, but by the very transnational network his persecutors sought to dismantle.

**Figure 1.**
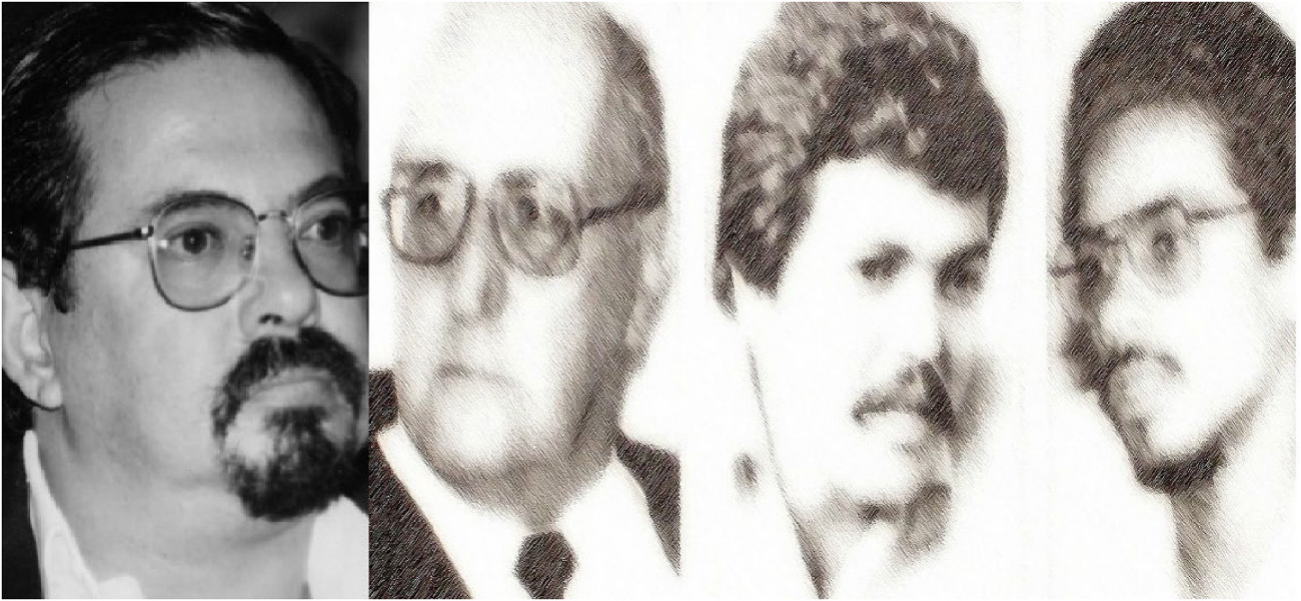
The faces nof those who were assassinated during the bloody August of 1987 in Medellin, Colombia. From left to right – Dr. Pedro Luis Valencia, Hector Abad-Gomez, Leonardo Betancur, Luis Felipe Velez.

Herein lies the paradox at the heart of this history. The campaign to atomize and destroy this community of leftist medical practitioners became the catalyst for its fortification. The violence intended to silence a movement instead amplified its cause, breathing new life into the international solidarity that would ensure its survival. What emerged as a regional tragedy was transformed into a powerful testament to transnational resistance – ALAMES, a regional network of leftist intellectualism in health, morphed into a unique case of leftist humanitarianism.

This article reevaluates the history of humanitarianism through a grassroots international network at the margins of Latin American anti-communist. Its theoretical foundation is anchored on three key ideas: ‘humanitarianism’ as a transnational practice alleviating suffering; ‘leftist politics’ as revolutionary and reformist movements challenging state and imperial authority; and ‘transnational solidarity’ as networks connecting these spheres.^6^ The main argument is that ALAMES transnationalism was a form of leftist solidarity in which suffering was politicized into a method for opposing oppressive regimes across the continent. While rooted in the humanitarian paradigms of the Cold War era, social medicine differs from other post-1970s iterations that relied on human rights discourse, operated through NGOs, and involved North–South power relations.^7^ Rather than liberal benevolence, the article shows how solidarity rooted in the collective struggle of social medicine leftism constituted a fundamentally different way of knowing and practising aid – one characterized by grassroots epistemology, South–South cooperation, and partisan commitments.

To fully understand this distinctive form of aid, it is crucial to trace the trajectory of Western humanitarianism as a narrative of significant transformation, with its rationale and practices persistently redefined. Historically, it is anchored in the principles formalized by the International Committee of the Red Cross (ICRC), emphasizing the ideals of neutrality, impartiality, and independence.^8^ The objective was to create an apolitical environment for medical relief during interstate conflicts, which would allow organizations to operate across zones without threatening parties. This shaped the institutional architecture of modern humanitarian efforts, as seen in the Geneva Conventions. While effective in some contexts, it was inadequate for Cold War-era ideological conflicts that pushed for the politicization of aid.

The postwar bipolar struggle transformed humanitarianism into a tool of statecraft, often employed for geopolitical objectives, such as supporting allied regimes and countering communist influence, as seen in the US's Alliance for Progress in Latin America.^9^ This instrumentalization contravened neutrality and fostered widespread cynicism about the motivations of Western aid. Furthermore, in response to the egregious violence of dictatorships and far-right regimes worldwide, the Cold War witnessed the emergence of a radical counter-movement that rejected impartiality.^10^ The 1971 founding of *Médecins Sans Frontières* (MSF), inspired by the Biafran War, introduced a ‘new humanitarianism’ that claimed neutrality was equivalent to complicity in the face of dominance and oppression.^11^ This ideological schism created a lasting rift in humanitarianism: classicists, emphasizing access and neutrality, versus solidarists, prioritizing justice and advocacy. The latter, linked with leftist ideologies in the anti-communist landscape of Latin America, relied on the European legacy of rights to document terrorism, condemn abuses, and reinforce exile narratives.^12^

In contrast to traditional leftist humanitarianism, conceived in response to the regional epistemicide of the left, Latin American social medicine reaches beyond the Western-centred developmentalist narrative. It embodies a unique form of collective action that instrumentalized hostility into an ideology style addressing the social basis of health and disease in ways absent in the human rights discourse.^13^ Its practitioners mobilized the adversities of state terrorism to collaboratively establish an autonomous health discourse grounded in resistance, which moved away from intergovernmental aid and legal language. Solidarity was driven by a shared sense of pain and dignity, effectively dissolving the distinction between donor-recipient. It elucidated a form of transnational support that functioned outside the scope of prevailing humanitarian models through the circulation of people, ideas, and resources.

The article draws on oral history interviews with key ALAMES actors and examines the association's archives in Colombia and Argentina. It also uses secondary sources analysing socio-material conditions and narratives of violence, including institutional interviews and secondary publications. This article proceeds in two sections. The first discusses the Latin American social medicine network, highlighting its leftist roots in critique of postwar preventive medicine, and situates social medicine within Cold War regional politics and leftist humanitarian practices. The second section explores repression and solidarity, focusing on the lived experiences of the Condor Operation in the Southern Cone, the peak of anti-communism. It assesses social medicine's impact on the history of leftist humanitarianism and its lasting legacy.

The Latin American social medicine embodies critical thinking across various political and philosophical perspectives, unified by a focus on the relationship between society and health. While historically eclectic, post-Second World War tensions shifted a significant portion of the field into a distinctly leftist political movement, whereby ALAMES is notable for its transnational endeavours.^14^ The association was founded at the Third Latin American Seminar on Social Medicine in Ouro Preto, Brazil (1984), sponsored by the Pan-American Health Organisation (PAHO), in honour of pioneer Juan Cesar Garcia – an Argentinian scholar, paediatrician, and sociologist.^15^ Although a mere milestone in a long-standing process, ALAMES exemplifies leftist intellectualism in health amidst anti-communist adversity.

Following Churchill's infamous Iron Curtain speech, regions outside major power centres became testing grounds for projects designed to sway local interests towards contested versions of modernity. In Latin America, Western foreign policy employed ideas about state-building, political centralization, and cultural hegemony to advance an anti-communist agenda.^16^ The health sector adopted paternalistic programmes that were dependent on foreign technologies, experts, and international funding, using military-style strategies inspired by US warfare to eradicate tropical diseases.^17^ Its rhetoric promoted benevolent knowledge transfer from central hubs to underdeveloped countries – a form of Western exceptionalism that proliferated through higher education and the influence of local sociopolitical elites.^18^ The emphasis on elites was pivotal, as it marginalized the working class and the most vulnerable communities, namely, the societal sectors that had been the focus of populist movements in earlier leftist revolutions.

US soft power aimed to uproot communist influence by improving population well-being, a key concern for regional states. Its focus on health made it vital for development during the 1960s anti-imperialist uprisings, which tackled rural and urban poverty, labour exploitation, and land monopoly.^19^ At the forefront of US efforts was the 1961 Charter of Punta Del Este, which inaugurated the Alliance for Progress (AfP) under John F Kennedy. Mainly a response to the Cuban Revolution, the AfP was a comprehensive foreign aid strategy encompassing healthcare, housing, social infrastructure, and education.^20^ Programmes were developed in collaboration with international organizations, including the Rockefeller Foundation (RF), the Milbank Memorial Fund (MMF), and PAHO, while meticulously controlling their design, deployment, and evaluation. Initially met with enthusiasm, the AfP nearly exhausted all funding and political support by the Nixon Administration.

Despite setbacks, the persistence of changes in health policies represents a remarkable achievement of the AfP's anti-communist discourse.^21^ Funding schemes helped standardize medical education and public health across Latin America by introducing the US model of health developmentalism: the so-called ‘Preventive Medicine’ or preventivism. Originating from 1940s reforms, preventivism gained prominence through the 1952 RF-funded Conference on Preventive Medicine in Colorado Springs, followed by WHO meetings in France, Sweden, and the UK.^22^ Rooted in technocratic methods, the model was first introduced in Latin America via negative reports on regional medical education by US-based international health organizations. According to different authors, the reports paralleled ongoing US interventionism by fuelling fear over the spread of communism in higher education.^23^

Post-Second World War social medicine origins critique preventivism, opposing quantitative health planning, development technocracy hierarchy, and functionalist social theory of emerging international health.^24^ Among the voices, Sergio Arouca, a pioneer of social medicine and leader of the Brazilian Sanitarismo movement, is often central in the debate. His discourse began addressing the PAHO seminars in Preventive Medicine and Medical Education in Chile (*Viña del Mar*, 1955) and Mexico (*Tehuacan*, 1956),^25^ which made preventivism the focus of medical curriculum reforms in the region.^26^ Multiple events stemmed from this initial effort, which changed the landscape of clinical training.^27^ Garcia explained:

Latin American medical education was considered scientifically *backward, separate from prevention, undisciplined, and methodologically anachronic*. The PAHO, the Rockefeller Foundation, the Milbank Foundation, and the Point IV Program joined efforts to *correct* these deficiencies. The PAHO took charge of ‘modernizing medical education.’ The Rockefeller Foundation standardized medical education in areas that were isolated from the urban centers. The Milbank Foundation focused on the social sciences in health. And Point IV incorporated social scientists, particularly anthropologists, in its plans for action.^28^

This critique highlights social medicine's view on preventivism as a policy-driven imposition of an epistemological and pragmatic divide between local and global public health. The split enabled international organizations to execute a successful campaign that colonized knowledge production and healthcare practices, steering regional care towards market-oriented biomedicine. Consequently, social medicine arose as a movement that opposed developmentalism's master-slave and veered towards the vindication of local idiosyncrasies.

Numerous studies show resistance to US-style curriculum reforms at public universities, where staff viewed these changes as suppressing ongoing protests on the ground.^29^ In Colombia, for example, the implementation of the preventive model required the creation of new medical schools in peripheral regions of the country, such as Cali and Manizales. In these areas, recently recruited younger personnel facilitated this transition owing to their prior training at US public health departments and their independence from the centralized influence of traditional institutions located in Bogota and Medellin.^30^

For Arouca, preventivism rested on the dominance of Western technology and technocratic approaches at the expense of engaging with the fundamental socioeconomic, political, and cultural processes in health.^31^ Western medicine reduced health to mere biological determinants, which legitimized one-size-fits-all interventions, flattening the complexities of real life. Euphemisms such as ‘standardization’ and ‘efficiency’ enabled the drive to universalize the singular US approach. The belief that ‘apolitical’ science and technology could tame nature was, according to Arouca, a strategy that aided the expansion of preventivism in Latin America. In this manner, social medicine characterized preventivism as a means to expand markets for techno-scientific research – mirroring the same short-term and curative approaches of clinical medicine but now aimed at the earlier stages of disease.

Building on the success of preventive medicine in Latin America, the PAHO initiated an evaluation of its impact, as well as the prerequisites for its sustained implementation. In 1968, the organization's Department of Human Resources appointed Juan Cesar Garcia, then a research associate at the Harvard Department of Public Health, to spearhead the inaugural comparative study of Latin American institutions, which culminated in his renowned publication ‘*La Educacion Medica en la America Latina*.’^32^ Co-financed by the MMF, Garcia's research explored medical schools in the region that promoted preventivism at the core of the teaching, expanding PAHO's database from 100 in the mid-1960s to 178 a decade later.^33^

Although Garcia's research was relevant for understanding the prevalence of preventive medicine across Latin America, the scholar employed the project as a foundation for a considerably more intricate initiative: the creation of the Latin American Social Medicine Network, which was a precursor to ALAMES. Conceived initially to enhance the integration of critical social theory into health research and education, the network rapidly evolved into a transnational nexus of humanitarian support for health professionals amid the wave of state violence in the 1970s.^34^

By the late 1960s, as conservative elites reinforced stricter anti-communist policies aligning with US security strategies, preventivism emerged as another contentious arena in the Cold War Western front. Among the newly established departments of preventive medicine and public health, physicians driven by political opposition to the developmentalist paradigm became prime targets of an international Red Scare.^35^ Reputations were damaged, political participation was stigmatized, collective actions were prohibited, associations and unions were dissolved, and colleagues faced dismissals. At the zenith of state terrorism, social medicine scholars encountered persecution, detention, harassment, threats, kidnapping, exile, and assassination.

Enticed by the anti-developmentalist struggle, Garcia initially engaged with social medicine activists at medical schools in a spirit of camaraderie. However, as violence escalated, state terror shifted his focus towards deploying strategies safeguarding the well-being of colleagues. Building upon the resources and database provided by PAHO, Garcia utilized the institutional map of preventive medicine schools to connect political dissenters and collaboratively develop a cartography of transnational humanitarianism. Under the guise of exploring innovative perspectives on health, the scholar facilitated the circulation of persecuted practitioners, radical ideas, and healthcare practices to ensure epistemic survival. The emerging social medicine network gathered a variety of traditions advocating for the study of sociopolitical and economic determinants of health, progressively constructing a health epistemology rooted in collective action and solidarity.

Figure 2.

**Figure 2.**
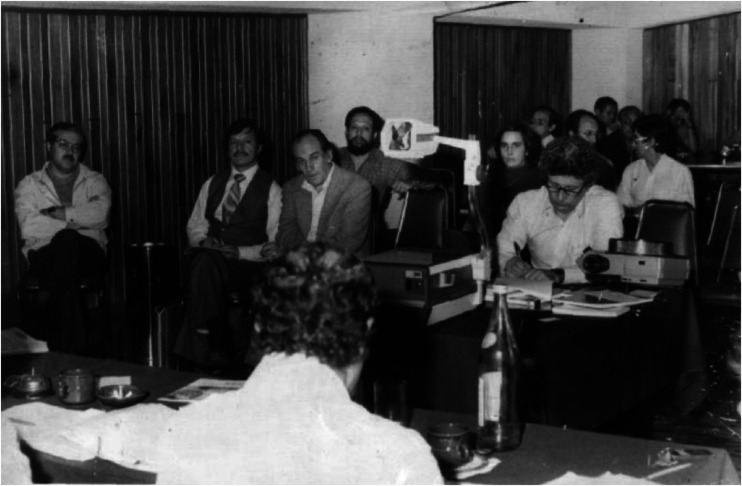
Conference talk at the second Latin American social medicine seminar 1983 (Cuenca, Ecuador). *Source:* Waitzkin et al. ‘Social medicine then and now.’ American Journal of Public Health 91, no. 10 (2001): 1595.

At PAHO, Garcia identified strategies to strengthen the Latin American social medicine network through international conferences, network meetings, and academic workshops.^36^ The 1972 First Latin American Seminar on Social Medicine at *Universidad de Cuenca* in Ecuador marked a milestone, showcasing social medicine's international voice and addressing epistemological concerns in the region for the first time.^37^ The meeting consolidated the group, which remained in close contact throughout the following decade, organizing 7 workshops and 14 seminars. Though focused on academia, the continuous interaction kept alive the flame of reciprocity, solidarity, and support in times of dictatorships. Against the backdrop of a renewed conservative PAHO in the mid-1970s, Garcia's work became the ‘*mecenas de la izquierda’* or protector of leftist ideologies within the multilateral agency.^38^

To that end, the network founded two postgraduate programmes. With support from the Kellogg Foundation (KF) and the United Nations Development Program (UNDP), social medicine pioneers at PAHO led the creation of the Institute of Social Medicine at the *Universidade do Estado do Rio de Janeiro* in 1973 (UERJ, Brazil), as well as the Masters in Social Medicine at *Universidad Autonoma de Mexico-Xochimilco* in 1975 (UAM-X, Mexico).^39^ The endeavours are a testament to the pragmatism of leftist networks in Latin America, exploiting the anti-communist agendas of Western organizations for their purposes, through covert roles such as advisers, officers, or even leadership positions.

Both academic centres exemplify social medicine humanitarianism. UERJ helped establish the *Centro Brasileiro de Estudos de Saúde* (*Cebes*) in 1976, a key hub for critical health studies via journals like *Saúde em Debate.*^40^ The staff at the centre were linked to Garcia's network, belonging to the Brazilian *Sanitarismo* movement, which harboured political dissenters during the dictatorship, including Sergio Arouca, Emerson Merhy, Sonia Fleury, Mario Testa and Jairnilson Paim-Silva.^41^ Alongside Brazilian efforts, Garcia commissioned Salvadorian cardiologist, public health expert, and ALAMES co-founder Maria Isabel Rodriguez to develop a social medicine degree in Mexico. She chose the country deliberately, connecting with Ramon Villareal, former PAHO head and UAM-X Chancellor, who was also a supporter of anti-developmentalist struggle.^42^ Villareal, a physician and social medicine pioneer, played a key role in the social medicine network by hiring Garcia for the PAHO study on medical education, guiding his Latin American journey. The Mexican postgraduate programme supported those fleeing terrorism by funding staff positions and scholarships for international students from hostile countries. Notable beneficiaries include social medicine leaders like Hugo Mercer, José Carlos Escudero, Saul Franco, and Jaime Breilh.

Following the tumultuous 1960s,^43^ the US heightened its focus on more extreme control measures, supporting military coups that employed tactics targeting individuals and groups even tangentially associated with left-leaning ideologies.^44^ Social medicine members were inevitably caught in the fire. By the time ALAMES was established in the early 1980s, the transnational network was predominantly recognized for its support of exiles rather than for broadening its conceptual framework. The critical juncture emerged from the primary anti-communism campaign, The Condor Operation: a meticulously crafted international strategy orchestrated by the US defence apparatus aimed at unifying and synchronizing efforts to eradicate communism throughout the Southern Cone.^45^ The operation commenced with the Bolivian coup led by General Rene Barrientos in 1964. It interconnected right-wing military regimes under the collective ‘national security’ policy across Brazil, Uruguay, Chile, Paraguay, and Argentina. For social medicine representatives, the Condor Operation obscured the distinctions between political and academic spheres, rapidly extending state terrorism into public universities and scholarly environments. Edmundo Granda, Ecuadorian physician, PAHO representative, and ALAMES honorary member explained:

A fundamental change in local politics characterised the end of the 1960s and the early 1970s: the demise of a utopian democracy…. The dictatorships and state of emergency became the norm in Latin America as a form of ‘bourgeoisie democracy.’ The left-wing comrades who managed to save their lives sought refuge in countries where they could still breathe. The social medicine pioneers of the time … had to flee upon learning about the massive assassination of leaders…. Popular movements were swept away overnight while the holocaust of left-wing politics grew into nightmarish proportions.^46^

Granda differentiated between two forms of democracy in Latin America during that period. The first, described as ‘utopian’, was supported by the social medicine network and aligned with the populist movements that gained prominence in the early twentieth century. This approach addressed enduring conflicts related to agricultural reforms, labour standards, and social care.^47^ The second, rooted in Western dominance, concealed social inequalities by imposing a vision of socioeconomic and political order centred around multilateralism with the US. Rather than equal and reciprocal relationships, Granda contended for the colonial continuity in this approach, whereby the liberalization of markets predominantly benefited the elites alone.^48^

For Granda, the most remarkable feature of the time was the unprecedented force through which the bourgeoisie took over the continent for the sake of a Westernized social order. Traditionally, the Condor wave of dictatorships is presented against the belligerent backdrop of left-leaning ideologies as these grew woven into the matrix of National Liberation Armies and other *guerrillas*.^49^ Though partly accurate, social medicine scholars argue that this narrative distorts the reality of anti-communism by obscuring the disproportional intensity deployed against non-military grassroots movements.^50^ The Chilean case, which includes physician and social medicine pioneer Salvador Allende, illustrates the point.^51^

Democratically sworn into office in 1970, Allende ascended to prominence through a distinguished career in politics, advocating a ‘socio-medical’ conception of the population and pioneering socialist policies, including the establishment of the first national healthcare system in the region.^52^ Allende's interest in the social conditions underlying medical disorders inspired numerous social medicine practitioners, including Howard Waitzkin – primary care physician, emeritus professor of the Department of Sociology and Health at the University of New Mexico, and ALAMES honorary member. According to Waitzkin, the prospect of electing a socialist government in Latin America was considered inconceivable, given that the US historical approach to the region was fundamentally imperialistic within an otherwise bipolar global context.^53^ Indeed, Allende advocated for a popular-front government with a strong anti-capitalist stance, condemning the market enterprise as callous, disdainful, and exploitative.^54^

The social medicine collective during the era of Allende was distinguished by its emphasis on action concerning the health implications of social issues, including wages, food sovereignty, housing conditions, clothing, and sanitation facilities. While practitioners such as Benjamin Viel and Gustavo Molina Guzman concentrated their efforts on the national healthcare system, other scholars opted to maintain a connection between health problems and macro-level societal issues. Initiating from medical concerns, reformers examined their underlying social processes, highlighting the political economy of underdevelopment and the persistent monopoly over natural resources to interpret conditions such as Tuberculosis and Typhus as ‘social diseases’. Molina Guzman was actively involved in the social medicine network, contributing to the medical literature that circulated the region in times of dictatorship.

On 11 September 1973, the *coup d'état* was initiated. The Presidential Palace *La Moneda* was subjected to bombing by the National Air Force and was subsequently overtaken by heavy artillery under the command of General Gustavo Pinochet, the de facto leader. Arturo Jiron, Minister of Health under Allende and the last individual to see him prior to the cabinet's surrender, disclosed that the president committed suicide, following the tradition of José Manuel Balmaceda, the reformist president of Chile who killed himself in 1891 rather than surrender to a military coup.^55^ Following the success of the Pinochet coup, comprehensive structural reforms aimed at liberalizing the market from state regulation and reforming the social welfare system were enforced through coercive measures, establishing Chile as a pioneering experiment in neoliberal policies.^56^

Colleagues and sympathizers of Allende's politics were detained or exiled. Maria Isabel Matamala, physician, public health professional, and founder of the ALAMES feminist section, suffered the era's inhumane conditions. A member of the *Movimiento de Izquierda Revolucionaria* (Revolutionary Leftist Movement, MIR) since 1967, she managed children's healthcare programs in Santiago at the onset of the coup.^57^ As all government sectors ceased operations, Matamala adhered to MIR protocols and concealed herself within a clandestine lifestyle. She severed all affiliations with the central committee and maintained a low profile, regularly changing residences and creating new identities. ‘(MIR members) knew that, if we were caught, life would be over … we committed to revealing no information about our own regardless of the consequences’, she explained.^58^ Matamala managed to survive this way for a few months, that is, until she was apprehended while moving between locations.

Matamala was first detained at the infamous camp *Villa Grimaldi* and moved to the prison centre *Cuatro Alamos.* Managed by the *Direccion de Inteligencia Nacional DINA* (National Intelligence Directorate, a form of Gestapo during the dictatorship), the scholar was subjected to gruesome forms of physical violence and sexual abuse. She explained: ‘They took us to what we called ‘*parrillas’* (grills in Spanish) to torture us. Electrocute us. Or hang us from the ceiling for a beating. This was a festivity to the guards – all sexual, verbal and physical aggression caused laughter and enjoyment.’^59^ Prisoners lived in small cabins under utter darkness and poor insulation, forced to take turns to sleep at night due to the limited space. Countless atrocities have been documented at these sites, with testimonies attesting widespread fear, uncertainty, humiliation and pain.^60^ Waitzkin was among the first to circulate information on the tragedies occurring in Chile through the prestigious New England Journal of Medicine*,* which quickly gaining international interest.^61^ As a result, he was subjected to public shaming and death threats from the regime's sympathizers, and was prevented from entering Chile thereafter.

Building upon these historical considerations, the general assertions about solidarity within leftist humanitarian social medicine find a concrete, lived expression in the case of Argentina, particularly concerning the rationale articulated in the alternative curricula at their centres. Following the CIA's repressive agenda, General Jorge Rafael Videla staged a coup against Maria Estela Martinez de Peron in 1976, who was the acting leftist president following the death of her husband.^62^ The dictatorship established a *Junta Militar* that initiated a wave of violence prolonged by successive regimes until re-democratization in 1983. Alicia Stolkiner, a professor of clinical psychology at the Universidad de Buenos Aires and ALAMES former general coordinator, exemplifies the dialectics between critical perspectives and authoritarianism.

To be clear, Stolkiner is a prime example of how the social medicine network provided refuge, intellectual community, and institutional infrastructure, thereby enabling the persistence and flourishing of the episteme in exile. Following tragic incidents with the regime, Stolkiner fled to Mexico in the late 1970s, where she engaged with psychotherapist colleagues Marie Langer, Silvia Bermann, and Ignacio Maldonado – all of whom were exiled and directing the *Comité de Solidaridad con el Pueblo Argentino* (Committee of Solidarity with the Argentine People, COSPA).^63^ The organization offered financial and emotional support during the initial years abroad and was instrumental in her subsequent involvement with ALAMES. Parallel to Stolkiner's adaptation, the triumphant Sandinista revolution in nearby Nicaragua motivated the UAM-X's Department of Social Medicine to establish a subdivision dedicated to mental health services supporting the most vulnerable.^64^ Through their close association with COSPA, Stolkiner was appointed to the UAM-X subdivision, where she engaged with colleagues in social medicine, representatives from PAHO, and the transnational network. Her participation at UAM-X was as crucial in establishing a postgraduate programme with critical psychology insights as the network was in safeguarding her well-being during exile.^65^

The specific nature of this alternative model of solidarity can be best understood through the scholar's life narrative. Born into a second-generation Menshevik family in Argentina, Stolkiner was raised under the banner of early socialist movements, developing a career that integrated psychoanalysis, Marxist political economy, and activism. Her formation occurred at the public *Universidad de Cordoba* during the 1969 ‘Cordobazo’, a period of student upheaval against the dictatorship of General Juan Carlos Organia.^66^ This revolutionary atmosphere led to her involvement in the anti-psychiatry movement, which introduced a range of critical theories, including Lacanian analysis and Althusserian structuralism, into mental health practices. She regarded leftist psychological theory as an essential counter-narrative to the right-wing ideologies prevalent during dictatorships, naturally aligning with them.

Following her graduation in 1972, the Argentinian Psychoanalysis Association (APA) underwent a significant schism. Two dissident groups, *Plataforma* and *Documento*, formed in opposition to military institutions, marking a political awakening among mental health professionals who had previously remained on the sidelines of social affairs.^67^ These groups, led by figures like APA founder Marie Langer, rejected the traditional ‘neutrality’ of the psychoanalytic profession and committed to using psychoanalysis in the service of the people by challenging societal structures that oppressed the working class.

Langer and colleagues consolidated Plataforma as a Marxist organization, subsequently renamed the *Coordinadora de Trabajadores de Salud Mental* (Movement of Mental Health Workers, CSTM), and founded research centres like the *Centro de Investigacion y Docencia* (Centre for Teaching and Research, CID).^68^ As outlined in their teaching charter (Figure 3), the principles linked psychiatric institutionalization with a capitalist ethos, reflecting the financial and political interests of emerging market monopolies. The centre directed its critique towards the institutional authority that enforced linear, mechanized, and commodified standards of diagnosis at the expense of more fluid, relational, and local systems of care – developing a blueprint of anti-imperialist pedagogy for social medicine abroad. Central to their practices, both CTSM and CID upheld the notion that healthcare should not be detached from political activism, urging members to pursue actions challenging the control exerted by the Junta Militar. Though fraught with flaws, the movement's impetus influenced young professionals like Stolkiner, who brought the Marxist approach into the Faculty of Psychology at the public *Universidad de Cordoba* and then UAM-X once in exile.^69^

**Figure 3.**
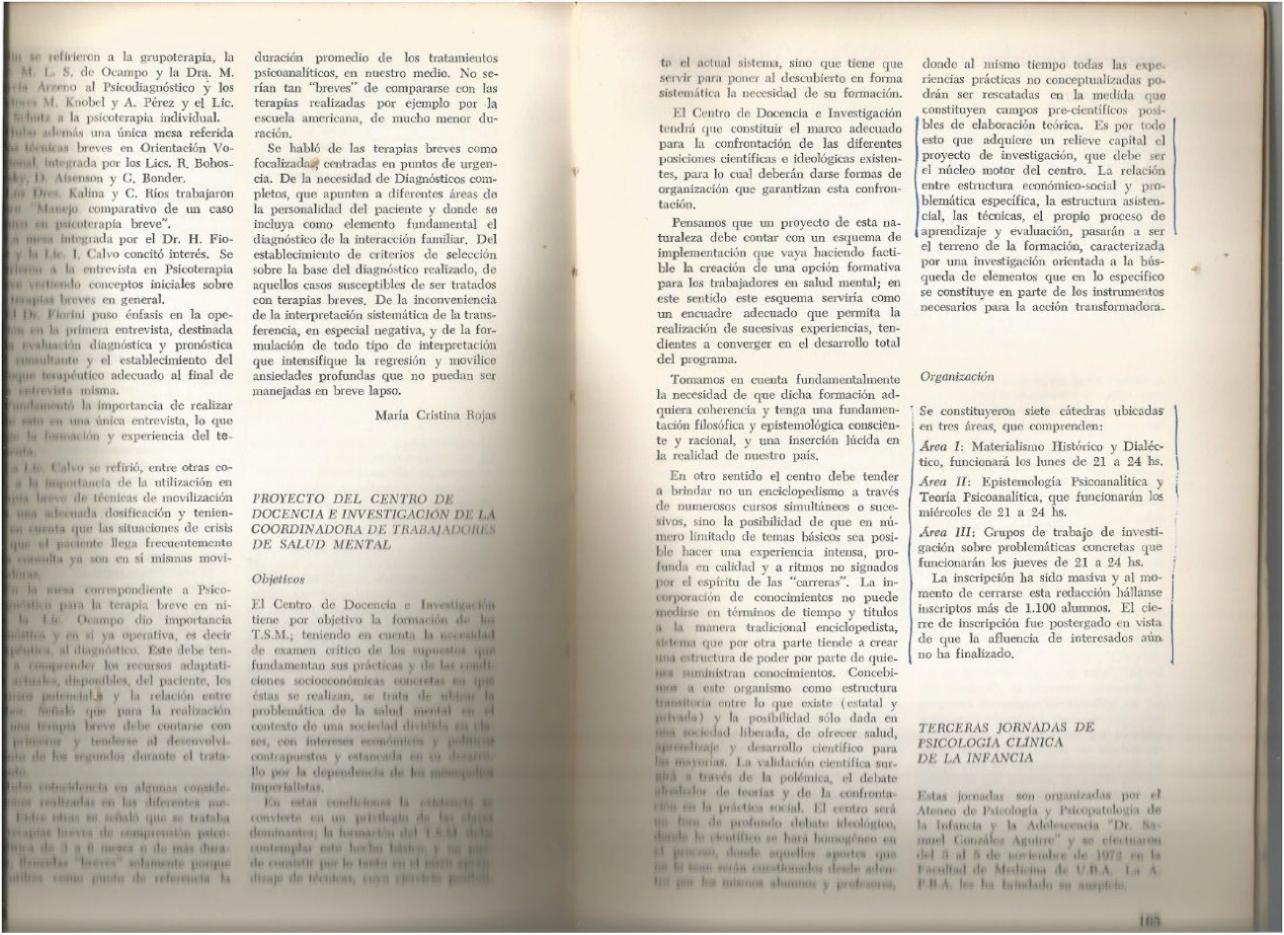
CID teaching charter published as ‘El Programa de Estudios del Centro de Docencia e Investigación de la Coordinadora de Trabajadores de Salud Mental (Argentina)’ Revista Argentina de Psicología, Año III, no. 12 (Junio 1972):164–165.

During Argentina's turbulent political landscape, Stolkiner faced significant persecution for her leftist political affiliations with the emerging Peronism and her involvement in platforms like the CID.^70^ In late 1975, she was detained and subjected to a brutal interrogation by the Argentine Anti-communist Alliance (Triple-A) in collaboration with the federal police at the *Camara del Terror* in Cordoba – one of the many infamous prison centres in the country. The interrogation included physical beatings, isolated confinement, and a simulated execution intended to extract information. Stolkiner explained: ‘Blindfolded, I was beaten pretty badly. They ordered to shoot me. They used to do what is known as ‘mock firing squad.’ I probably looked very calm to them, but in reality, I was so petrified that I couldn't utter a word. They kept pressing me to give them a name or reveal information to “save my life”.'^71^ Following her release, her home was raided by military police to further the sense of terror. These experiences underscore the political climate that shaped the practice of social medicine in Latin America.

Alicia Stolkiner's eventual exile from Argentina was directly precipitated by the severe persecution her family faced under the military dictatorship. Following the 1976 coup, the military regime, as part of its national security strategy, targeted sources suspected of funding the armed resistance, including the MacKentor Construction Company (MCC).^72^ The company, co-owned by the *Vaca Narvaja* and *Altamira Jofre* families, was accused of links to the *Montoneros* guerrilla group, leading to the sequestration of its assets and a violent crackdown. This reign of terror resulted in the assassination of members of the Vaca Narvaja family, followed by the abduction and presumed murder of Stolkiner's brother-in-law, Carlos Altamira Jofre, who had been forced into the company's leadership. The incident was the definitive trauma leading to Stolkiner's exile. She narrated the episode:

(Carlos) comes by my house and says: ‘Lend me your car, the one under your name. I have to return some paperwork, and my car is at the garage'…. At this time, to drive a borrowed car you had to have a signed authorisation from the owner. I feared he would be stopped and detained without this paper. So, I responded: ‘this is silly, I'm coming with you since the car is under my name'…. At that moment, I see a very dear friend walking towards the house…. I stepped out of the car, put Mariano (the son) in her arms to hug her, and I hear Carlos closing the car door and drive away. He never returned.^73^

The independent investigation, completed many years later, revealed that the brother-in-law managed to cross a nearby bridge where he parked the car to deliver paperwork. ‘At this point, a group of urban paramilitary known as ‘*Patotas’* arrived in a vehicle without plates and forced him into the trunk. Two witnesses claim to have seen him last at the *Centro de Detention Clandestina ‘La Perla’*, a detention centre known for extreme violence, torture, and the assassination of political leaders. All traces of Carlos are lost at this point and remain missing to this day. The following day, Stolkiner moved to her parent's home in Buenos Aires. Two weeks later, she was boarding a plane to Mexico. The deep sense of relief once the plane took off was overwhelming, she explained.

Figure 4.

**Figure 4.**
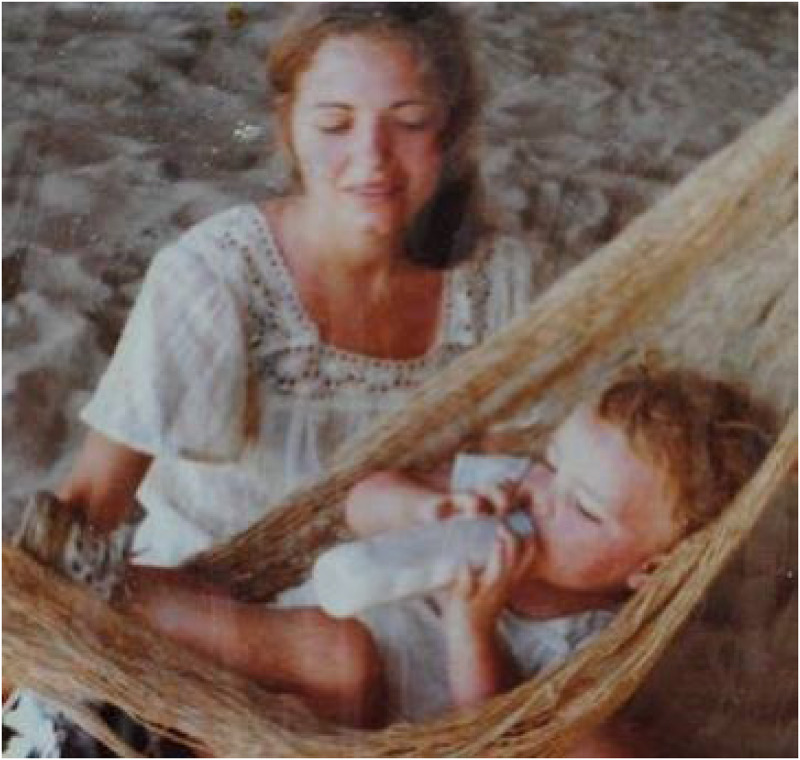
Alicia Stolkiner and son at the time of exile. *Source:* Stolkiner's personal archive.

Once linked to the transnational network, Stolkiner played a pivotal role in developing the curriculum for the UAM-X postgraduate degree in social medicine. The program was established in response to the rejection of the concept of ‘risk factors’ favouring a comprehensive understanding of the fundamental social processes that influence population health. Central to this approach is the perspective of health and disease as a dialectical process shaped by structural conditions and the individual's position within the class system – a framework directly aligned with the CID's hybrid methodology.^74^ Over time, aided by Stolkiner's contributions, the programme expanded its critique to include the political economy of health policy and the hegemonic nature of biomedical knowledge. Through the so-called ‘Social Determination model’, its more recent literature has concentrated on differentiating itself from the depoliticized Social Determinants of Health framework promoted by the WHO, upholding the activist spirit of Cold War resistance.^75^

Stolkiner's own work is shaped by the fruitful engagement with the social medicine network, championing the field of ‘collective mental health’ (*salud mental colectiva*), taught in the ALAMES social medicine course online.^76^ She argues against the decontextualized, individualizing, and often pathologizing frameworks of traditional psychiatry and psychology. Instead, she analyses how modes of production and social exclusion generate collective forms of mental distress. The epistemological tradition embodied by the UAM-X programme and Stolkiner's approach serves as a testament to how social medicine exile hubs operated as essential centres for the network's sustenance and intellectual advancement, where the circulation of people fostered discourses that rivalled ongoing human rights discussions.

Along with circulating people, anti-communist repression fuelled a covert movement of resources that reinforced an alternative form of leftist humanitarianism. Central to understanding this phenomenon is Susana Belmartino, a prominent public health historian, former director of the master's program at the Centre for Interdisciplinary Studies of the Public *Universidad Nacional del Rosario* (UNR) in Argentina, and ALAMES co-founder. Disrupted by General Juan Carlos Onganía's fascist regime, Belmartino left Argentina to complete her doctoral studies abroad, returning in the late 1960s to resume her role. However, the rise of paramilitary violence and the imminent *Junta Militar* in 1975 profoundly impacted scholarly circles, and Belmartino's work was deeply affected by the Latin American red scare. She explained:

In October of this year, as I was working in my first teaching position at the Faculty of Humanities in Rosario, the faculty received a note from ‘Triple A’ directed against thirty of its staff members … amongst these names was also mine. Given the immense violence of the time, we all decided to quit our positions. Not just that, we decided to disappear to our regions of origins, spread out in the country, for at least a few months until things calmed down. From this point onwards, I didnot work in history or any activity linked to academia.^77^

Safety concerns were undoubtedly well-placed. The region of La Plata was raided by the fascist collective *Concentracion Nacionalista Universitaria* (University Nationalist Concentration), who subjected leftist scholars to threats, tortures, disappearances, and murders, leading to the closure of several academic departments.^78^ ‘It is impossible to explain with words what happened and how we felt during this time – intellectuals were persecuted and slaughtered not merely with impunity but as exemplary cases of the consequences of our thinking’, remarked Eduardo Menendez, Argentinian social medicine practitioner.^79^ The anthropology department he helped establish at the *Universidad del Mar de la Plata* was entirely dismantled, with members either fleeing the country or being murdered.

Once blacklisted by anti-communist organizations, the risk to their lives was imminent. Belmartino left academia and survived in Rosario by maintaining a low profile, working temporary contracts and avoiding any ‘dangerous’ roles. ‘I entered a weird time in my life’, she explained, ‘I cannot tell you it was depression, but it was certainly a sense of resignation from academia.’^80^ By sheer coincidence, she was contacted by Carlos Bloch in 1978, a physician and social medicine scholar in Argentina. Belmartino joined Bloch's teams as a researcher in the history of medicine at the recently established *Centro de Estudio Sanitarios y Sociales* (Centre for Sanitary and Social Studies, CESS), the founding hub of the Journal *Revista Cuadernos Medico-Sociales* – paradoxically during the height of the Argentinian dictatorship in the late 1970s.^81^ Despite the wave of violence, the regime considered history of medicine neutral enough to pose no threat, enabling Belmartino's critical approach to preventivism.

At this time, Belmartino collaborated with PAHO officials and ALAMES co-founders Juan Cesar Garcia and Hesio Cordeiro in the initiative known as the ‘*Libro Gris’* (Grey Literature) ­– an academic clandestine network disseminating socialist literature throughout Latin America. As she articulated: ‘(The Libro Gris) was a series of photocopies of articles and books, mainly bibliographic references, on critical topics for the region that circulated during the dictatorship’.^82^ The connection with Brazil was robust due to Argentine scholars who migrated to other institutions, including *Cebes* and *Fiocruz* in Rio de Janeiro, supporting figures such as Adolfo Chorny, Mario Testa, and Mario Hamilton. According to Belmartino, ‘thinking health and politics’ under repression was possible only due to the transnational circulation of resources that allowed social medicine to be resilient and thrive in clandestine conditions. Indeed, much of the journal's content consisted of Spanish translations of Brazilian work by authors such as Sergio Arouca, Sonia Fleury, and Emerson Merhy.^83^

The methods employed for disseminating literature were, by necessity, covert and dependent on personal relationships, deliberately undocumented for security reasons. They encompassed a Samizdat-style reproduction, referring to the clandestine, manual copying and distribution of materials utilizing basic tools such as typewriters, carbon paper, and mimeographs.^84^ The hand-typed documents that comprised the fragmented ‘grey literature’ were subsequently circulated among readers in a chain, with each individual often producing their own copies and passing the imperfect versions to colleagues. This process established an unofficial, underground network of writers, typists, and distributors, each contributing to the dissemination of prohibited texts to a wider yet controlled audience. Trusted academics and students travelling internationally for the few authorized events or personal reasons acted as couriers, smuggling books, articles, and letters across borders within their luggage. International conferences became vital yet perilous venues for the exchange of these materials and ideas. Hubs like UAM-X and UERJ were instrumental in receiving, teaching, and re-exporting these critical ideas; also serving as sources of potent critiques and developing published work for circulation.^85^

Figure 5.

**Figure 5.**
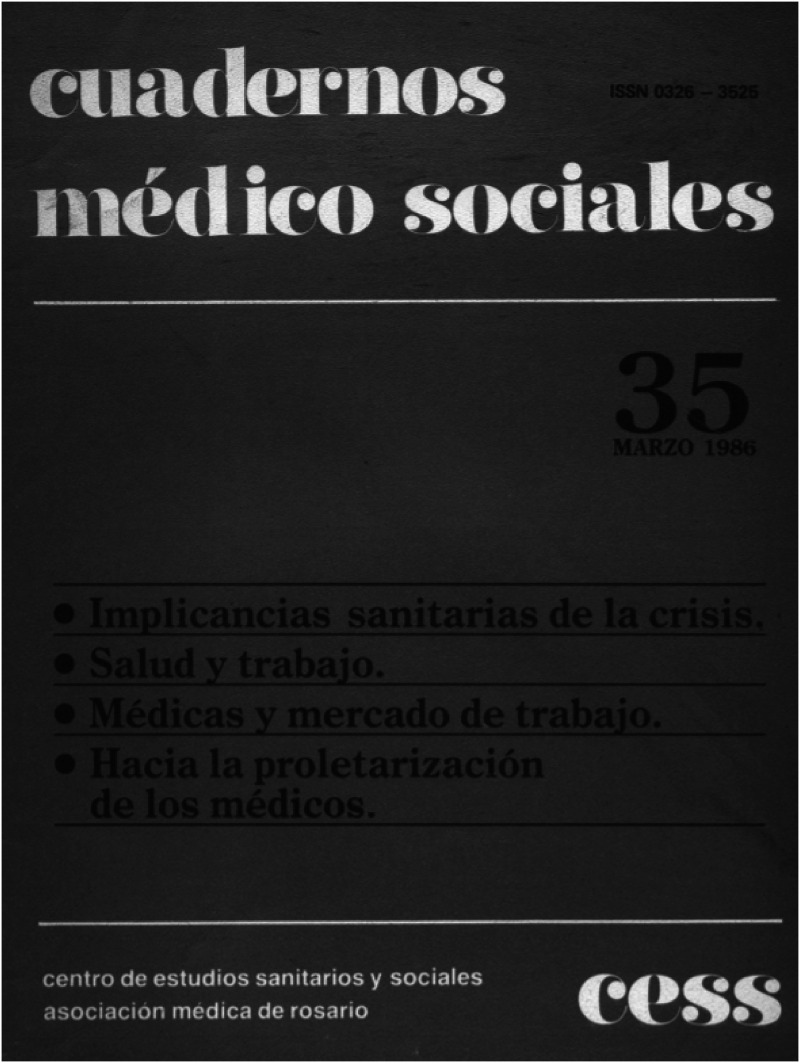
Cover of the Journal Cuadernos Medico-Sociales Volume 35, published in 1985. *Source:* Spinelli et al., ‘Cuadernos médico sociales’ História, Ciências, Saúde–Manguinhos, 24, no. 4 (2017), 877–895.

Though essential developments were published, authors were still forced to work using pseudonyms to avoid the dangers associated with critical thinking. According to Spinelli and colleagues, various publications by Bloch and Belmartino engaging with the decadent healthcare policies of the regime were published under the names of ‘Carlos Alarcon’ and ‘Susana Balmaceda.’ Juan Cesar Garcia utilized the pseudonym ‘Aureliano Mierr’, referencing the fictional character Aureliano Buendia from the Nobel prize-winning novel ‘*One Hundred Years of Solitude’* by writer Gabriel Garcia Marquez. Mario Testa, public health expert and ALAMES co-founder, skilfully combined the names of his children to come up with his fake name ‘Paulo Alexandre’. Jose Carlos Escudero at UAM-X used ‘Martin Sabelli’ to safely submit papers to journals, honouring the life of *guerrilla* members Maria Angelica Sabelli and Martin Fierro who the Argentinian regime had murdered during the so-called *Masacre de Trelew* (1972).

Scholarly research on leftist humanitarianism during the Latin American Cold War is anchored in the fundamental observation that the geopolitics of this era reconfigured the field into a arena of political contestation and advocacy. The existing literature divides this period into two distinct phases.^86^ First, a pre-1970s ‘politics of revolution’ that was intimately related with the emergence of National Liberation Armies, student movements, and social upheavals in response to the increasing incapacity to satisfy fundamental needs. This phase perpetuated a tradition of twentieth-century populist revolutions tackling land monopolies, labour exploitation, inadequate social safety nets, and the infringement of political rights for the working class. Second, a post-1970s ‘politics of emergency’ followed in the wake of military dictatorships, wherein leftist movements increasingly adopted less confrontational strategies that were centred on human rights to safeguard persecuted individuals. The transition into the second phase is marked by the Chilean *coup d'état*, which generated a wave of exiles who effectively secured international condemnation of the military *juntas* by exposing unprecedented levels of state violence and repression.^87^

Research on the post-1970s ‘new humanitarianism’ of the left was mainly geared towards examining the transnational networks of solidarity that supported the exiled population in Europe.^88^ As explained by Stites: ‘During the Cold War, as struggles for legitimate democracy and citizenship against dictatorship were often international in scale, human rights advocacy and transnational solidarity emerged as two of the most prominent modes of opposition to repressive regimes and to external intervention.’^89^ Given the rising prominence of human rights as a space for oppositional politics amidst state terrorism, most leftist collectives veered away from their Marxist roots and inhabited the uncharted territory in collusion with international NGOs. This move was also strategic as military regimes, preoccupied with retaining a minimum level of legitimacy in the eyes of the capitalist West, were driven to adhere to Euro-American agendas within which the human rights framework thrived.

Instead of criticizing developmentalist systems or delving deeper into the radicalization of the bipolar order, the new left solidarity preferred targeted legal strategies against state terrorism and institutional marginalization. Collectives used expert testimonies to pursue legal investigations, naming-and-shaming practices and actors, wrote letters to consulates and politicians to exert international pressure, put together booklets with illustrations and photographs of abuses, made public appearances at key international organizations (for example, the Organisation of American States, OAS, and the United Nations, UN), and wrote countless reports for renowned news outlets.^90^ Although impactful, this humanitarianism circumscribed the field within the epistemological coordinates of liberal and rights-focused approaches from Euro-American legacies. It reduced humanitarianism to individualist methods that risked perpetuating neocolonial narratives by situating the political agency of regional actors within Western paradigms – as if, outside Euro-US discourses, Latin American humanitarianism would not have flourished.

In contrast, Latin American social medicine presents a distinct perspective on humanitarianism that originated from a grassroots standpoint and was deeply embedded within the intellectual culture of public universities and South-South cooperation.^91^ Instead of adopting an internationalist language, social medicine concentrated on safeguarding knowledge producers, securing institutional safe havens (UAM-X, UERJ), and disseminating critical texts (the ‘*Libro Gris’*), thereby enabling the relational construction of a leftist epistemology that employs Marxist historical materialism to analyse the social basis of population health.^92^ Frameworks such as the Social Determination model, transnational solidarity, and covert activism unveil an approach that functioned independently of traditional human rights paradigms. They present an alternative historical narrative in which actors subverted Cold War adversities to pioneer innovative approaches towards health equity. In doing so, social medicine contests the notion that state institutions, multilateral organizations, or international NGOs based in the Global North are alone responsible for conceptualizing and practising humanitarianism, as depicted in the existing literature today.

Though social medicine reveals the limitations of a universalist definition of humanitarianism, prompting us to recognize solidarity as a distinct, politically grounded practice, the new leftist humanitarianism did not passively apply a legal framework to regional phenomena. Instead, actors imbued human rights with locally-constructed meaning, principles, and directives that expanded Euro-American perspectives into more concrete and applicable designs.^93^ This adaptation gave rise to a moral economy of support for the persecuted, a platform for the denunciation of abuses, a battlefield against state violence, and a blueprint to remember those lost and disappeared, involving institutions like Amnesty International, the International Court of Justice in The Hague, the Ford Foundation, and the International Commission of Jurists, amongst others.^94^

Through a common language, activism even transcended religious denominations in the Liberation Theology movement.^95^ Rooted in critiques of military repression, the long history of wealth and power maldistribution, and the systematic oppression of the laity, the clergy in countries like Argentina and Colombia mobilized followers into resistance through an interplay of theological claims and human rights language.^96^ Religious institutions exerted international pressure against regimes by filing habeas corpus petitions, publishing regular bulletins, establishing centres of solidarity (such as food banks, shelters, medical care, and spiritual support), and even engaging in secret dialogue with military authorities to negotiate the agenda.^97^ The hybridity of this collective was also instrumental for *guerrilla* movements, who were able to move through countries subversively thanks to the support of some of these institutions.

In this sense, practitioners of social medicine conceptualized health and disease, as being profoundly interconnected with the sociopolitical and economic processes associated with anti-communism, in a manner analogous to the ‘new humanitarianism’ concerning state violence. Nonetheless, rather than utilizing the international framework of conventions and declarations, scholars relied on their collective sense of suffering, shared indignation, and overlapping struggles to cultivate active support within their social capital. Following an alternative genealogy of collective action, social medicine actors within prominent US-based health organizations redirected their resources, infrastructure, and expertise – aimed initially at an anti-communist agenda – towards safeguarding, fostering intellectual development, and cultivating a health-related epistemology. Organizations such as PAHO became collaborators in facilitating leftist humanitarian efforts through centres like UERJ and UAM-X. This initiative benefited victims of the Condor Operation and beyond, thereby establishing a tradition that remains a viable alternative within the realm of decolonial historiographies and global health discourse.

This article has traced the trajectory of an alternative humanitarianism, one that flourished in the interstices of Cold War geopolitics. It has argued that the transnational network of Latin American social medicine, forged in response to brutal state repression, represents more than an unexamined case study. Its history is one of deliberate epistemic survival in the face of violent epistemicide. The campaign to ‘anular cerebros’ – to extinguish a leftist worldview by assassinating its thinkers – inadvertently catalysed a resilient, South-South network of solidarity dedicated to preserving and advancing that very knowledge. By safeguarding practitioners, securing institutional havens, and circulating clandestine texts, this movement's humanitarian practice was the act of keeping a critical epistemology alive. Its legacy, therefore, offers crucial insights that resonate far beyond the confines of Cold War Latin America.

The foundational critique of technocratic ‘preventivism’ by the social medicine movement offers a significant historical perspective for evaluating contemporary global health governance. The top-down health development models of the Cold War era, which simplified intricate social issues into technical solutions manageable by foreign experts, are reflected in current initiatives undertaken by prominent international organizations and private philanthropies.^98^ Analysing preventivism as an ‘apolitical’ instrument of US foreign policy provides a vital warning against present-day global health programmes that emphasize measurable outcomes and technological interventions while neglecting the political economy of disease and the structural violence underpinning health disparities. In more limited ways, this was replicated by the human rights approach of ‘new left’ solidarity in times of dictatorships. The legacy of ALAMES challenges these paradigms by advocating for a decolonial approach that affirms health as inherently linked to struggles for social justice, labour rights, and political sovereignty.

Furthermore, the story of epistemicide in Latin America resonates with disturbing clarity in the modern conflict landscape. The systematic targeting of physicians, academics, and public health leaders across the Southern Cone was a strategy intent on dismantling a community's capacity to heal, to think, and to resist. This tactic remains a central feature of contemporary warfare, visible in the deliberate destruction of universities, hospitals, libraries, and the assassination of intellectual and medical leaders worldwide – including the contemporary conflict in Gaza.^99^ The violent logic that targeted Hector Abad-Gomez in 1987 is the same that levels a university hospital today. To understand this is to recognize that the fight for survival is intrinsically linked to the fight for knowledge. The history of Latin American social medicine, therefore, is not just a regional story of leftist solidarity; it is a universal testament to the resilience of the collective and a vital blueprint for how communities can safeguard their knowledge and dignity in the face of annihilation.

## Notes

1 The Latin American Social Medicine Association, founded in 1984, aims to integrate social science and humanities into health research, education, and practice. It emphasizes that population wellbeing cannot be fully understood through biology alone, instead combining multiple disciplines to broaden research and theory. For details, see Borde, Elis; Mario Hernández. ‘Revisiting the social determinants of health agenda from the global south’. *Global Public Health* 14, no. 6-7 (2019): 847–62. Waitzkin, Howard; C. Iriart, A. Estrada, and S. Lamadrid. ‘Social medicine then and now: Lessons from Latin America’. *American Journal of Public Health* 91, no. 10 (2001): 1592; Adams, Vincanne; Dominique Behague; Carlo Caduff; Ilana Löwy, and Francisco Ortega. ‘Re-imagining global health through social medicine’. *Global Public Health* 14, no. 10 (2019): 1383–1400. On Franco’s Work and his commitment to humanitarianism, see *El Quinto, no matar: Contextos explicativos de la violencia en Colombia* (Santa Fe de Bogota, Colombia: Tercer Mundo SA, 1999).

2 The concept of epistemicide, broadly defined as the destruction, subjugation, or de-legitimization of a knowledge system, has become a cornerstone of decolonial theory and critical inquiry across the social sciences and humanities. Originally coined to describe a process more profound than mere disagreement or the natural fade-out of ideas, epistemicide suggests a violent, power-driven erasure of ways of knowing, especially those of non-Western, Indigenous, and subaltern communities. See, Santos, Boaventura de Sousa. *Epistemologies of the South: Justice Against Epistemicide* (Boulder, CO: Paradigm Publishers, 2014); Smith, Linda Tuhiwai. *Decolonizing Methodologies: Research and Indigenous Peoples*. Third edition (London: Bloomsbury Publishing Plc, 2021). https://doi.org/10.5040/9781350225282; Tiostanova, M.V., and Walter Mignolo. *Learning to Unlearn: Decolonial Reflections from Eurasia and the Americas* (Columbus, US: Ohio State University Press, 2012).

3 Founded in 1985, the UP originated from peace negotiations in the 1980s between President Belisario Betancourt's conservative government and FARC, a major guerrilla group. While uniting leftist leaders, the UP is mainly recognized for securing amnesty for FARC members to enter politics, despite their involvement in human rights violations and crimes. The agreement did not fully end FARC’s armed activities, creating a problematic situation where the group gained political legitimacy while continuing guerrilla warfare. For details, see Abraham, Jenaro, Kelsey Carlston, and Stacy Bondanella Taninchev. ‘Choking off Democratic Channels and the Resort to Violence: How the Political Genocide of Unión Patriótica (UP) Candidates Escalated and Perpetuated Colombia’s Armed Conflict’. *Small Wars & Insurgencies* 36, no. 1 (2025): 185–212. https://doi.org/10.1080/09592318.2024.2393496; Gomez-Suarez, Andrei. ‘The geopolitical situation of the genocidal destruction of the Patriotic Union (1985–2010)’. *Estudios políticos (Medellín, Colombia)* 43, no. 43 (2013): 180–204. Francesca Casafina. “Mass Violence, Memory and Paths for Justice. The Colombian Case of Unión Patriótica in Light of Inter-American Court’s Judgment’. *Scienza e Politica* 35, no. 68 (2023): 109–24. https://doi.org/10.6092/issn.1825-9618/17742.

4 UP members and elected congressmen were systematically attacked and assassinated throughout the 1980s in a phenomenon otherwise known as the 'Genocidio de la Union Patriotica' (UP Genocide). The latest report by the National Centre for Historical Memory documents at least four thousand UP members kidnapped, 'disappeared', or assassinated - including two presidential candidates Jaime Pardo Leal and Bernardo Jaramillo. In 2014, the Colombian Office of the Attorney General declared the genocide a 'crime against humanity’ where the political elite, state military, and elites articulated in the events as part of a decade-long plot to 'impede the rise of leftist's movement that emerged as a political alternative to the national conflict’. For details, see Steele, Abbey. *Democracy and Displacement in Colombia’s Civil War*. 1st ed. (Ithaca: Cornell University Press, 2017). https://doi.org/10.1515/9781501709753; Dudley, Steven. *Walking Ghosts: Murder and Guerrilla Politics in Colombia* (New York, NY: Routledge, 2005). https://doi.org/10.4324/9780203499566; Gómez Gómez, Nathaly. ‘Una aproximación a la construcción de memoria(s) de la Unión Patriótica en Colombia desde los testimonios de sobrevivientes’. *Etcétera*, no. 7 (2020). Full Castaño interview found in Mauricio Aranguren. *Mi Confesion: Carlos Castaño Revela Sus Secretos* (Bogota D.C., Colombia: Editorial Oveja Negra, 2001).

5 Abad-Gomez was renowned in Colombia for establishing the first public health department at the Universidad de Antioquia. His legacy in academia and social medicine remains significant, having led the regional Committee for the Defence of Human Rights and strongly supporting leftist-progressive ideas and policies. Likewise, Betancourt was a public health activist and a member of the same Committee, notably a leader of the leftist movement *Firmes* (founded in the late 1970s as an intellectual collective, securing seats in the Senate during the 1980 elections). For details, see Zea Bustamante, Luis Emilio. ‘Héctor Abad Gómez como educador popular. Un acercamiento a su vida, obra y discursos’. *Revista de la Facultad Nacional de Salud Pública* 35, no. 2 (2017): 179–85. https://doi.org/10.17533/udea.rfnsp.v35n2a02.La No Ficción (2020). *Ya no tengo miedo.* Available at: https://open.spotify.com/episode/1bVqFaGlJGnp5cfIpztLe5?si=Eqg7IF9vS7STqI-YX2JxOg (accessed 05 March 2025). Hector Abad Faciolince. *El Olvido que Seremos* (Bogota D.C., Colombia: Alfaguara, 2018).

6 Hatzky, Christine, and Jessica Stites Mor. ‘Latin American Transnational Solidarities: Contexts and Critical Research Paradigms’. *Journal of Iberian and Latin American Research* 20, no. 2 (2014): 127–40. doi:10.1080/13260219.2014.939121; Pirker, Kristina. ‘Activismo transnacional y solidaridad, de Cuba a Centroamérica’. *Revista de la Red de Intercátedras de Historia de América Latina Contemporánea* 4, 7 (2017): 120–138; Grimaldi, Anna & Marty, Samira. ‘Introduction to solidarity politics: The (re)activation of European-Latin American solidarities’. *Alternautas, 11,* no.1 (2024). https://doi.org/10.31273/an.v11i1.1692

7 Denéchère, Yves. ‘Eastern Europe - A New Field of Humanitarian History’. *Eastern Journal of European Studies* 5, no. 2 (2014): 5–11; Vargha, Dóra. *Polio Across the Iron Curtain: Hungary’s Cold War with an Epidemic* (Cambridge: Cambridge University Press, 2018) https://doi.org/10.1017/9781108355421; Iacob, Bogdan C. ‘A Babel in Bucharest: Third World Students in Romania, 1960s-1980s’. *Cahiers Du Monde Russe* 63, no. 3/4 (2022): 669–90. https://doi.org/10.4000/monderusse.13289. Mark, James, and Paul Betts, eds. *Socialism Goes Global: The Soviet Union and Eastern Europe in the Age of Decolonisation* (Oxford: Oxford University Press, 2022)

8 After the 19th-century Battle of Solferino, the classical or ‘Dunantist’ humanitarian model was based on the core principles above. While admirable, it reflected European state system assumptions about sovereignty and conflict. Its main practice was discreet negotiations with sovereign states to access prisoners of war and civilians, assuming clear distinctions between combatants and non-combatants, and political and humanitarian spheres. For details, see Moorehead, Caroline. *Dunant’s Dream: War, Switzerland and the History of the Red Cross* (London: HarperCollins, 1998); Barnett, Michael N. *Empire of Humanity: A History of Humanitarianism* (Ithaca: Cornell University Press, 2011); Weiss, Thomas George. *Humanitarian Intervention: Ideas in Action* (Cambridge, England: Polity Press, 2016).

9 The Cold War fundamentally altered the landscape of humanitarian action. See Fassin, Didier. *Humanitarian Reason: A Moral History of the Present*. 1st ed. Berkeley: University of California Press, 2011. https://doi.org/10.1525/9780520950481; Rieff, David. *A Bed for the Night: Humanitarianism in Crisis* (London: Vintage, 2002); Kevin Hartigan, ‘Matching Humanitarian Norms with Cold, Hard Interests: The Making of Refugee Policies in Mexico and Honduras, 1980–89’, *International Organization* 46, no. 3 (January 1, 1992): 709–30, https://doi.org/10.1017/S0020818300027879. Details on the US Alliance for Progress in Rabe, Stephen G. *The Most Dangerous Area in the World : John F. Kennedy Confronts Communist Revolution in Latin America* (Chapel Hill: University of North Carolina Press, 1999).

10 See, Neier, Aryeh. *The International Human Rights Movement: A History*. 2nd ed. (Princeton, United States: Princeton University Press, 2020). https://doi.org/10.1515/9780691200996; Keck, Margaret E, and Kathryn Sikkink. *Activists beyond Borders: Advocacy Networks in International Politics*. 1st ed. (Ithaca, N.Y: Cornell University Press, 1998). Markarian, Vania. ‘Uruguayan Exiles and Human Rights: From Transnational Activism to Transitional Politics, 1981–1984’. *Anuario de Estudios Americanos* 64, no. 1 (2007): 111–40. https://doi.org/10.3989/aeamer.2007.v64.i1.35.

11 This movement, explored in the writings of MSF figures like Rony Brauman, rejected the ICRC's tradition of silent diplomacy. They championed the principle of *témoignage*, or bearing witness. For details, see Brauman, Rony. ‘Médecins Sans Frontières and the ICRC: Matters of Principle’. *International Review of the Red Cross* 94, no. 888 (2012): 1523–35. https://doi.org/10.1017/S1816383113000283.

12 Latin America during the Cold War became a primary theatre for these competing models. The region was characterized not by conventional interstate wars, but by counter-insurgency campaigns, military dictatorships, and civil wars fueled by superpower patronage. US foreign policy objectives directly shaped the contours of violence and the corresponding humanitarian crises, particularly in the cases of the so-called ‘Condor Operation’ and the dirty wars in Central America. Organizations like Amnesty International often overlapped and sometimes conflicted with the work of providing material relief and documenting the abuses. For details, see Sanahuja, José Antonio, and Augusto Delkáder–Palacios. ‘Ayuda, Hegemonía y Poder: EEUU y La Configuración de La Agenda Global Del Desarrollo’. *Revista de Fomento Social*, no. 300 (2021): 301–27. https://doi.org/10.32418/rfs.2021.300.5030; Susan D. Burgerman, ‘Mobilizing Principles: The Role of Transnational Activists in Promoting Human Rights Principles’, *Human Rights Quarterly* 20, no. 4 (1998): 905–23, https://doi.org/10.1353/HRQ.1998.0035; Berger, Mark T. ‘Managing Latin America: US Power, North American Knowledge and the Cold War’. *Journal of Iberian and Latin American Research* 2, vol. 1 (1996): 41–63. doi:10.1080/13260219.1996.10431803. On the Latin American Cold War, see Grandin, Greg. *The Last Colonial Massacre: Latin America in the Cold War* (Chicago; London: University of Chicago Press, 2011).

13 This argument reiterates parallel ones made about the regional social medicine, against the assumptions of its origins rooted in the European counterparts i.e., Rudolf Virchow’s hygienic movement. For details, see Carter, Eric D. *In Pursuit of Health Equity: A History of Latin American Social Medicine* (Chapel Hill, US: University of North Carolina Press, 2023); Carter, Eric, and Marcelo Sánchez Delgado. ‘A Debate over the Link between Salvador Allende, Max Westenhöfer, and Rudolf Virchow: Contributions to the History of Social Medicine in Chile and Internationally’. *História, Ciências, Saúde--Manguinhos* 27, no. 3 (2020): 899–917. https://doi.org/10.1590/s0104-59702020000400011; Breilh, Jaime. *Critical Epidemiology and the People’s Health* (Oxford, United Kingdom: Oxford University Press, 2020).

14 Details on the postwar social medicine in Latin America, see Fonseca, Sebastian. ‘Latin American Social Medicine : The Making of a Thought Style’ (PhD dissertation for Dpt Global Health and Social Medicine, King’s College London, 2020); Escorel, Sarah. *Reviravolta na Saúde: Origem e Articulação do Movimento Sanitário* (Rio de Janeiro, Brazil: Editora Fiocruz, 1999); Birn, Anne-Emanuelle, Carles Muntaner, and Zabia Afzal. ‘South-South Cooperation in Health: Bringing in Theory, Politics, History, and Social Justice’. *Cadernos de Saúde Pública* 33, no. suppl 2 (2017): e00194616. https://doi.org/10.1590/0102-311×00194616.

15 Juan Cesar Garcia was diagnosed with advanced-stage Lymphoma and underwent treatment in the early 1980s. He died from the disease in 1983, shortly after the Second International Seminar in Social Medicine celebrated in Cuenca, Ecuador. He left behind a great legacy that significantly contributed to the revival of social medicine in Latin America in the poswar era. Details in Galeano, Diego, Trotta, Lucia, and Spinelli, Hugo. ‘Juan Cesar Garcia and the Latin American Social Medicine Movement: Notes on a life trajectory’. *Salud Colectiva.* 7, no. 3 (2011): 285–315. Duarte-Nunes, Everardo. ‘Juan Cesar Garcia: Social medicine as project and endeavor’. *Ciencia & Saude Coletiva.* 20, no. 1 (2015): 139–144. Breilh, Jaime, ‘Componente de Metodología: La Construcción del Pensamiento en Medicina Social’. Chap. II in Franco, Saul, Duarte-Nunes, Everardo, Breilh, Jaime, Laurell, Cristina, *Debates en Medicine Social* (Quito, Ecuador: Organización Panamericana de la Salud, 1991). On the establishment of ALAMES, see ALAMES. ‘Acta de Ouro Preto: Constitucion de la Asociacion Latinoamericana de Medicina Social (1984)’. *Medicina Social.* 4, no. 4 (2009): 263–264.

16 Cueto, Marcos. *Cold War, Deadly Fevers: Malaria Eradication in Mexico, 1955–1975* (Washington DC: The Johns Hopkins University Press, 2007); Harmer, Tanya. ‘The Cold War in Latin America’. In *The Routledge Handbook of the Cold War*, edited by Artemy M. Kalinovsky and Craig Daigle, 1st ed. (London, UK: Routledge, 2014): 133–48. https://doi.org/10.4324/9781315882284-14; William A. Booth, ‘Rethinking Latin America’s Cold War’, *The Historical Journal* 64, no. 4 (September 1, 2021): 1128–50, https://doi.org/10.1017/S0018246X20000412.

17 Examples of health developmentalism in the Cold War, see Randall M. Packard, ‘Malaria Dreams: Postwar Visions of Health and Development in the Third World’, *Medical Anthropology* 17, no. 3 (May 1, 1997): 279–96, https://doi.org/10.1080/01459740.1997.9966141; Socrates Litsios, ‘Malaria Control, the Cold War, and the Postwar Reorganization of International Assistance’, *Medical Anthropology* 17, no. 3 (May 1, 1997): 255–78, https://doi.org/10.1080/01459740.1997.9966140; R. Magalhães and Marcos Chor Maio, ‘The “Good Neighbor Policy for Yellow Fever”: Cold War, the Aedes Aegypti Eradication Program of the United States and International Cooperation in Health in the Americas’, *Historia* 41 (January 1, 2022), https://doi.org/10.1590/1980-4369e2022026.

18 Corinna R. Unger, ‘The United States, Decolonization, and the Education of Third World Elites’ (Palgrave Macmillan, London, 2011), 241–61, https://doi.org/10.1057/9780230306486_13; Gilman, Nils. *Mandarins of the Future: Modernization Theory in Cold War America* (Baltimore: Johns Hopkins University Press, 2003). Details on the US ideological struggle in the Cold War see Martin J. Medhurst, ed., *Cold War Rhetoric: Strategy, Metaphor and Ideology* (East Lansing: Michigan State University Press, 1997). Scott Lucas, *Freedom’s War: The American Crusade against the Soviet Union* (New York: New York University Press, 1999). Rana Mitter and Patrick Major, eds., *Across the Blocs: Cold War Cultural and Social History* (London: Frank Cass, 2004).

19 Nicole Sackley, ‘The Village as Cold War Site: Experts, Development, and the History of Rural Reconstruction’, *Journal of Global History* 6, no. 3 (November 1, 2011): 481–504, https://doi.org/10.1017/S1740022811000428; Thomas Robertson, ‘Cold War Landscapes: Towards an Environmental History of US Development Programmes in the 1950s and 1960s’, *Cold War History* 16, no. 4 (October 1, 2016): 417–41, https://doi.org/10.1080/14682745.2014.950238.; Mulliken, Otis in *The United States and International Health: Hearings before a Subcommittee of the Committee on Interstate and Foreign Commerce, House of Representatives, 84th Congress, February 8 and 9, 1956* (Washington, DC: US Government Printing Office, 1956): 33–34. James Stevens, ‘Welcoming Address’, in *Industry and Tropical Health: Proceedings of the First Tropical Health Conference Sponsored by the Harvard School of Public Health, 8–10 December 1950* (Boston, US: Harvard School of Public Health and International Council for Tropical Health, 1950): 11–15.

20 For details, see Field, Thomas C. *From Development to Dictatorship: Bolivia and the Alliance for Progress in the Kennedy Era* (Ithaca: Cornell University Press, 2016); Chester Bowles, ‘Why Foreign Aid?’ *Department of State Bulletin* 48 (1963): 777–84; Taffet, Jeffrey F. *Foreign Aid as Foreign Policy: The Alliance for Progress in Latin America* (New York: Routledge, 2007); Galeano, Eduardo. Open Veins of Latin America: Five Centuries of the Pillage of a Continent, (New York: C. Belfrage, 1997 [1973]): 6–7.

21 Birn, Anne-Emanuelle, and Raúl Necochea López. *Peripheral nerve: health and medicine in Cold War Latin America* (London: Duke University Press, 2020); Theodore M. Brown, Marcos Cueto, and Elizabeth Fee, ‘The World Health Organization and the Transition from “International” to “Global” Public Health’, *American Journal of Public Health.* 96, no. 1 (2006): 62–71; Anne-Emanuelle Birn, *Marriage of Convenience: Rockefeller International Health and Revolutionary Mexico* (Rochester: Rochester University Press, 2006).

22 On the Colorado Spring Conference, see Clark, KG. ‘Preventive medicine in medical schools; report of Colorado Springs Conference November 52’. *Journal of medical education* vol. 28, no. 10 vol. 2 (1953): 1–123; Molina, Gustavo. ‘La Medicina Preventiva en la Educacion Medica’. *Boletin de la Oficina Sanitaria Panamericana (OSP)* 36, no. 4 (1954) 442–453. Wolf, George. ‘Preventive Medicine 1952–1964’. *The Milbank Memorial Fund Quarterly.* 42, no. 4 (Part 1) (1964) 9–21. Details on Preventivism in Mota, Andre, Schraiber, Lilia, Carvalho, Jose Ricardo. ‘The “paulista way”: Building Collective Health in the state of Sao Paulo, Brazil’. *Interface (Botucatu)*. 21, no. 60 (2017). 5–11; Birn, Anne-Emanuelle. *Marriage of Convenience: Rockefeller International Health and Revolutionary Mexico* (Rochester, US: Boydell & Brewer, 2006). https://doi.org/10.1017/9781580466646; Krieger, Nancy. *Epidemiology and the People’s Health: Theory and Context*. 1st ed. (Oxford: Oxford University Press, 2011). https://doi.org/10.1093/acprof:oso/9780195383874.001.0001.

23 Nicole Pacino. ‘National Politics and Scientific Pursuits: Medical Education and the Strategic Value of Science in Postrevolutionary Bolivia’. In *Peripheral Nerve*, (Duke University Press, 2020) https://doi.org/10.2307/j.ctv14t48qj.7; Cueto, Marcos. ‘International Health, the Early Cold War and Latin America’. *Canadian Bulletin of Medical History* 25, no. 1 (2008): 17–41. https://doi.org/10.3138/cbmh.25.1.17; O’Brien, Thomas F. *Making the Americas: The United States and Latin America from the Age of Revolutions to the Era of Globalization* (Albuquerque: University of New Mexico Press, 2007); Cottam, Martha L. *Figures and Intervention: U.S. Policies in Latin America* (Pittsburgh: University of Pittsburgh Press, 1994). https://doi.org/10.2307/jj.3177132.

24 Along the lines of preventivism, the functionalist social theory emerges from US-based scholars like Talcott Parsons, which treated society as a stable system that decontextualized diseases. Social medicine scholars like Asa Cristina Laurell and Juan César García critiqued it as (a) ahistorical, ignoring the underlying trajectory and structures of social class that determine why certain populations are exposed to disease while others are protected; (b) reductionist, as it flattened the complex social phenomena to a simplistic chain of individual risk factors, obscuring the ‘social determination’ of health; and c) Conservative, focusing on individual behaviour modification, diverting attention from the need for profound structural changes to address the root causes of inequality and ill-health. For details, see Duarte-Nunes, E. ‘Trayectoria de la medicina social en America Latina: Elementos para su configuración’. Chap. I in Franco, S. et al, *Debates* (Quito, 1991); 17–137; Jairnilson S. Paim, and Naomar de Almeida Filho. ‘Saúde Coletiva: Uma “Nova Saúde Pública” Ou Campo Aberto a Novos Paradigmas? Collective Health: A “New Public Health” or Field Open to New Paradigms?’ *Revista de Saúde Pública* 32, no. 4 (1998): 299–316. https://doi.org/10.1590/S0034-89101998000400001; García, Juan César. ‘La Medicina Estatal en América Latina (1880–1930)’. *Revista cubana de salud pública* 42, no. 1 (2016): 143–75.

25 The original reports on PAHO Seminars on Preventive Medicine and Education are found in Pan-American Health Organization. ‘Participantes en el Seminario de Enseñanza de la Medicina Preventiva celebrado en Tehuacán, Puebla, México, del 23 al 28 de abril de 1956’. *Boletín de la Oficina Sanitaria Panamericana*. 41, no. 1 (1956); and PAHO, *Seminarios sobre la Enseñanza de Medicina Preventiva*. 1955; Viña del Mar, Chile. 1956; Tehuacán, México (Washington: Oficina Sanitaria Panamericana, n. 28, 1957).

26 Arouca, Sergio. *O Dilema Preventivista: Contribuição para a Compreensão e Crítica da Medicina Preventiva* (PhD Dissertation: Universidad de Campinas UNICAMP, Sao Paulo, 1975). Further details in Arouca, S. (2013). ‘Democracia é saúde’. Paper presented at the *VIII National Health Conference in Brazil. 1986.* Available at: https://youtu.be/-_HmqWCTEeQ (accessed on 04 February 2025); Duarte-Nunes, Everardo. ‘La salud colectiva en Brazil: Analizando los procesos de institucionalizacion’. *Salud Colectiva.* 12, no. 3 (2016). 347–360. Mota, Andre, Schraiber, Lilia, Carvalho, Jose Ricardo. ‘Developmentalism and preventivism at the root of Collective Health: teaching reforms and the creation of Medical Schools and Departments of Preventive Medicine in Sao Paulo state, Brazil, 1948–1967’. *Interface.* 22, no. 65 (2018). 337–48. doi: https://doi.org/10.1590/1807-57622017.0810.

27 A very comprehensive list of seminars, publications, health programs, departmental projects, reports, books, discussions and other activities is recorded by Arouca (ibid., 1975:134). These include the First Seminar in Medical Education in Colombia (1955), the First and Second Conferences of Latin American Faculties of Medicine in Mexico (1957) and Uruguay (1960), and the meeting at UNESCO’s Centro de Cooperacion Cientifica para America Latina in Venezuela (1960). Further details in PAHO. *Enseñanza de la Medicina Preventiva y Social – 20 Años de Experiencia Latinoamericana* (Washington D.C.: PAHO Scientific Pub. No. 234, 1976). On the involvement of the Kellogg Foundation in medical education, see Chávez, Mario, Glen Cuthbert. *Un hemisferio unido: un tributo de la Fundación Kellogg a la Organización Panamericana de la Salud en la celebración de su centenario* (Michigan: W.K. Kellogg Foundation; 2003).

28 Garcia, Juan Cesar. ‘Juan Cesar Garcia Entrevista a Juan Cesar Garcia’. *Medicina Social.* 2, no. 3 (2007): 154.

29 Some works unfolding the ‘activeness’ of local population in assimilating, adapting, transforming or resisting international health programs include de Almeida, Marta. ‘Combates sanitários e embates científicos: Emílio Ribas e a Febre Amarela em São Paulo’, *História, Ciências, Saúde—Manguinhos* 6, no. 3 (2000):577–607; Obregón, Diana. *Batallas contra la lepra: Estado, medicina y ciencia en Colombia* (Medellín: Banco de la República de Colombia, 2002); Hochman, Gilberto, and Armus, Diego. Eds *Cuidar, controlar, curar: Ensaios Históricos sobre Saúde e Doença na américa latina e caribe* (Rio de Janeiro: Editora FIOCRUZ, 2004).

30 Téllez, Marlin, and Emilio Quevedo. ‘The Birth of a Ministry of Public Health in Colombia, 1946–1953: Cold War, Invisible Government and Asymmetrical Interdependence’. *História, Ciências, Saúde--Manguinhos* 29, no. 2 (2022): 461–80. https://doi.org/10.1590/S0104-59702022000200009; Birn, A.-E. ‘Backstage: The Relationship between the Rockefeller Foundation and the World Health Organization, Part I: 1940s–1960s’. *Public Health (London)* 128, no. 2 (2014): 129–40; https://doi.org/10.1016/j.puhe.2013.11.010; Banta, H D. ‘Flexner and Medical Education in Colombia’. *Journal of Medical Education* 47, no. 11 (1972): 879–85; Luis Aurelio Ordoñez. *Universidad del Valle 60 años: 1945–2005* (Cali, Valle del Cauca: Editorial Universidad del Valle, 2007); Quevedo, Emilio; Leonardo Palacios-Sanchez, Maria Camila Nuñez-Gomez; Maria Catalina Sanchez-Martinez. *El Renacer de una Ilusion: Historia de la Facultad de Medicine y de la Escuela de Medicina y Ciencias de la Salud de la Universidad del Rosario, 1969–2019* (Bogota, D.C., Colombia: Editorial Universidad del Rosario, 2021).

31 Arouca. *O Dilema Preventivista* (Sao Paulo, 1975), 66.

32 Details in PAHO, ‘Primer informe sobre la enseñanza de la medicina preventiva y social en las escuelas de medicina de la America Latina’. *Educación Médica y Salud*. 3, no. 2 (1969) 132–55; and PAHO. ‘Las escuelas de salud pública en la América Latina’. *Educación Médica y Salud*. 3, no. 3 (1969) 167–217. On the reports and outputs, see Garcia, J. C. *La Educación Médica en la América Latina* (Washington D.C.: PAHO Scientific Publication No. 255, 1972). Preliminary information found in García, Juan César. ‘Investigación sobre la enseñanza de la medicina preventiva y social en las escuelas de medicina de la América Latina’. *Educación Médica y Salud* 1, no. 4 (1966) 339–343.

33 PAHO’s mapping of institutions was established in the 1960s via the PALTEX program. PALTEX stands for the Expanded Textbook and Instructional Materials Programs, conceived in 1966 at the 17^th^ Pan-American Sanitary Conference and linked to the PAHO-led Latin American and Caribbean Center for Health Science Information (BIREME). The program aggregated high-quality medical education resources accessible to affiliated institutions across the region. Details in Barros da Silva, Márcia Regina, Ferla, Luis Antonio, Claramonte Gallian, Dante. ‘Uma “biblioteca sem paredes”: história da criação de BIREME’. *História, Ciências, Saúde – Manguinhos* 13, no. 1 (2006) 91–112; and Rodriguez, M. I. Entrevista con Dra. Maria Isabel Rodriguez. Interview for the *XIII ALAMES Conference at El Salvador.* UISP El Salvador January 12th, 2016*.* Available at: https://youtu.be/ULKyCwXPLR0 (accessed on 10 March, 2025). On Garcia’s work expanding the database, see Rodriguez, Maria Isabel. ‘El Estudiante de Medicina – Su distribucion en las America 1971–1972’. *Educacion Medica y Salud.* 8, no. 4 (1974). 360–89.

34 Here, critical social theory is code for Marx’s historical materialism applied to health, used to analyse the political economy of health, class analysis to explain unequal outcomes, and a critique of commodification to challenge market-driven approaches emerging in healthcare by the late 1970s–early 1980s. For details, see Breilh, Jaime. *Epidemiología crítica: Ciencia Emancipadora e interculturalidad.* 1ª. Ed. (Buenos Aires, Argentina: Lugar Editorial, 2003); Débora Tajer, ‘Latin American Social Medicine: Roots, Development during the 1990s, and Current Challenges’, *American Journal of Public Health* 93, no. 12 (December 1, 2003): 2023–27, https://doi.org/10.2105/AJPH.93.12.2023; Emily E. Vasquez, Amaya Perez-Brumer, and Richard Parker, ‘Social Inequities and Contemporary Struggles for Collective Health in Latin America’, *Global Public Health* 14 (May 20, 2019): 777–90, https://doi.org/10.1080/17441692.2019.1601752.

35 The anti-communist crusade known as McCarthyism in the United States extended beyond domestic politics, forming a core part of US foreign policy and reaching into the seemingly neutral areas of science and health. This ‘Medical McCarthyism’ aimed to marginalize, delegitimize, and actively suppress health paradigms that connected illness to social and economic structures, branding them as subversive and communist. As the main intellectual alternative to the US-endorsed biomedical and preventive approaches, social medicine became a significant target of this political campaign project. For details on Medical McCarthyism, see Merlin Chowkwanyun, ‘“The Neurosis That Has Possessed Us”: Political Repression in the Cold War Medical Profession’, *Journal of the History of Medicine and Allied Sciences* 73, no. 3 (July 1, 2018): 255–73, https://doi.org/10.1093/JHMAS/JRY015. On the political tensions underlying social medicine, see Thomas Pierret, ‘Social Medicine and the Social Sciences in Latin America: Conceptual Tensions for the Transformation of Public Health in the 20th Century’, *Oxford Research Encyclopedia of Global Public Health*, May 24, 2023, https://doi.org/10.1093/acrefore/9780190632366.013.333

36 Breilh, Jaime. *Critical Epidemiology and the People’s Health*. 1st ed. (United Kingdom: Oxford University Press, 2020). Almeida-Filho, N., & Silva-Paim, J. ‘La crisis de salud publica y el movimiento de la salud colectiva en america latina’. *Cuadernos Medicos Sociales, 75*, no 1 (1999): 5–30; Z. Betancourt, C. Hermida, H. Noboa & M. Rodriguez (Eds.), *Salud y vida: Volumen 1* (Quito, Ecuador: ALAMES, Universidad de Cuenca, CONASA, OPS/OMS, 2009); Rojas, F., & Marquez, M. *ALAMES en la memoria: Selección de lecturas.* (1st ed.). (La Habana: Cuba: Editorial Caminos, 2009).

37 As Galeano and colleagues explained: ‘the incipient group took an explicitly critical position regarding the functionalist theoretical framework that prevailed in the sociological analyses of health at that time’. In Galeano, Diego et al., ‘Juan Cesar Garcia and the Latin American Social Medicine Movement’ *Salud Colectiva.* 7, no. 3 (2011): 296. For more details, see Duarte-Nunes, Everardo, ed. *Ciencias Sociales y Salud en America Latina: Tendencias y perspectivas* (Montevideo, Uruguay: Organizacion Panamericana de la Salud – CIESU, 1986). Granda, E. ‘Algunos elementos sobre el desarrollo de la salud pública en América Latina’. Chap. in Z. Betancourt et al. *Salud y vida: Volumen 1* (Quito, Ecuador: ALAMES, Universidad de Cuenca, CONASA, OPS/OMS, 2009).

38 Interview with Mario Rovere, July, 2018.

39 Duarte-Nunes, E. ‘Trayectoria de la medicina social’ in Franco, S. et al, *Debates* (Quito, 1991); 48; and Duarte-Nunes, Everardo. ‘Ciencias sociales, salud y creación de instituciones de investigación en Brasil’. Interview by Galeano, D. for the seminar series *Oral History of the Social Medicine and Collective Health.* (Centro de documentacion Pensar en Salud, at the Instituto de Salud Colectiva, Universidad de Lanus Argentina. August 20th, 2015). Available at: https://youtu.be/hbyxLejgPcU?si=pkTZ5sBVuqawXyoS (accessed on 03 February, 2025).

40 Paim-Silva, Jairnilson. *Saúde política e reforma sanitária* (Salvador: CEPS-ISC; 2002); Fleury S, Bahia L, Amarante PDC, eds. *Saúde em Debate:* fundamentos da Reforma Sanitária (Rio de Janeiro: Editora CEBES, 2007); Amarante, Paulo, Frizon Rizzotto, Maria Lucia, and Costa, Ana Maria. ‘The history of a movement: Saúde em debate magazine and brazilian health reform’. *Ciencia & Saude Coletiva* 20, no. 7 (2015): 2023–9.

41 Sanitarismo was the label given to the group of left-leaning healthcare professionals in Brazil that, throughout the 70s and 80s, advocated for the end of the military dictatorship, the return to democracy, and major reforms to the national healthcare system. The success of the movement is marked by the inclusion of healthcare as a fundamental human right enshrined in the national constitution (1988), and realized in the new healthcare system SUS (Sistema Único de Saúde). The SUS is a publicly funded (tax-based) model that guarantees access to healthcare services and resources at the point of entry, with no premiums, no out-of-pocket payments, or other financial burdens constraints. For details, see Merhy, Emerson. *A saude publica como politica: Um estudio de formuladores de políticas* (Sao Paulo, Brazil: Editora Hucitec, 1992); Silva-Paim, Jairnilson. ‘A reforma sanitária brasileira e o sistema único de saúde: Dialogando com hipóteses concorrentes’. *Physis, Revista De Saude Colectiva* 18, no. 4 (2008): 625–644

42 Rodriguez, Maria Isabel. ‘Trayectoria María Isabel Rodríguez: Aportes en ciencia, educación y salud’. Interview at channel *El Salvador Ahora TVES CANAL 10*, 2019. Available at: https://youtu.be/tK8fzuVIPBo?si=dFXA40KfUBGJieZp (accessed on 3 February, 2025).

43 The 1960s is regarded for the extremely proliferous growth in politics and philosophy marked by the exponential expansion of decolonial movements, human rights upheavals, the new left in public universities, and student protests across the globe. In Latin America, the decade marked a new chapter in leftist belligerence, with the success of the Cuban Revolution, Che Guevara’s *foquismo,* the proliferation of National Liberation Armies, and the growth of armed agrarian collectives. For details, see Escobar, Arturo. *Encountering Development: The Making and Unmaking of the Third World* (Princeton: Princeton University Press, 2011). https://doi.org/10.1515/9781400839926; Marchesi, Aldo. *Latin America’s Radical Left: Rebellion and Cold War in the Global 1960s* (Cambridge University Press, 2017). https://doi.org/10.1017/9781316822968; Samantha Christiansen, and Zachary A. Scarlett. *The Third World in the Global 1960s (Protest, Culture and Society, Volume 8)*. 1st ed. Vol. 8. (New York, NY: Berghahn Books, 2012). https://doi.org/10.1515/9780857455741; Harmer, Tanya, Alberto Martín Álvarez, *Toward a Global History of Latin America’s Revolutionary Left*. 1st ed. (Gainesville: University of Florida Press, 2021). https://doi.org/10.2307/j.ctv1hqdj9q.

44 Gill, Lesley. *The School of the Americas: Military Training and Political Violence in the Americas*. (Durham: Duke University Press, 2004). https://doi.org/10.1515/9780822386001; Lessa, Francesca. *The Condor Trials: Transnational Repression and Human Rights in South America*. 1st ed. (New Haven: Yale University Press, 2022); Grandin. *The Last Colonial Massacre*; Zanchetta, Barbara. ‘Between Cold War Imperatives and State-Sponsored Terrorism: The United States and “Operation Condor”’. *Studies in Conflict and Terrorism* 39, no. 12 (2016): 1084–1102. https://doi.org/10.1080/1057610X.2016.1159069.

45 Rovere, M. Conversatorio 7: Foro interculturalidad y salud (Mario Rovere y Nila Heredia). Focus groups and Seminar Discussion for the *Introductory Course to Latin American Social Medicine* carried out by ALAMES. 2018. Available at: https://youtu.be/Sd3h5_anTOQ (accessed on 20 January, 2025); McSherry, J. Patrice. ‘Operation Condor and Transnational State Violence against Exiles’. *Journal of Global South Studies* 36, no. 2 (2019): 116–46; Lessa, Francesca, and Lorena Balardini. ‘No Safe Haven: Operation Condor and Transnational Repression in South America’. *International Studies Quarterly* 68, no. 2 (2024). https://doi.org/10.1093/isq/sqae035; Kornbluh, Peter. ‘Operation Condor: State-Sponsored International Terrorism’. In *The Pinochet File*. (New York, United States: The New Press, 2004).

46 Edmundo Granda, ‘Algunos Elementos sobre el desarrollo de la Salud Publica en America Latina’. In Z. Betancourt et al. *Salud y vida* (Quito, 2009):44

47 Responding to US-led ‘Pan-Americanism’ in the early twentieth century, these movements corresponded to forms of regional identity rooted in a shared colonial legacy and ongoing structural injustices. See, Birn, A., & Muntaner, C. ‘Latin American social medicine across borders: South-South cooperation and the making of health solidarity’. *Global Public Health, 14, no.*6–7 (2019), 817–834. doi:10.1080/17441692.2018.1439517; Meade, Teresa A. *A history of modern Latin America: 1800 to the present*. Second edition. ed. (Chichester, UK; Malden, MA: Wiley-Blackwell, 2010); and Grandin, Greg, Joseph, Gilbert eds. *A Century of Revolution: Insurgent and Counterinsurgent Violence during Latin America's Long Cold War* (Durham and London: Duke University Press, 2010).

48 For details on neo-imperialism in Latin America, see Grandin, Greg *Empire’s Workshop: Latin America, the United States, and the Rise of the New Imperialism* (New York: Metropolitan, 2006); Garcia, Roberto, Taracena-Arriola, Arturo. *La Guerra Fria y el Anticomunismo en Centroamerica* (Guatemala City, Guatemala: FLACSO, 2017); Joseph, G. M, and Daniela Spenser. *In from the cold: Latin America’s new encounter with the Cold War* (London: Duke University Press, 2008).

49 *Guerrillas* in inspired by the Cuban Revolution and Ernesto ‘Che’ Gevara’s *foquismo* include the *Tupamaros* in Uruguay, *Montoneros* in Argentina, *Guerrilla de Ñancahuazú* in Bolivia, the *FARC* in Colombia, and the *Sendero Luminoso* in Peru.

50 Belligerence based on a long-standing history of land dispossession, labour exploitation, forced displacement, and agrarian conflicts. For details, see Solheim, Håvar. ‘Review of Latin American Guerrilla Movements. Origins, Evolution, Outcomes’. *European Review of Latin American and Caribbean Studies*, no. 115 (2023): 5–5. https://doi.org/10.32992/erlacs.11001: Alberto Valencia Gutierrez. *La Violencia años cincuenta contada por sus victimas: Los Archivos de la Comision Investigadora* (Cali, Valle del Cauca: Universidad del Valle, 2021); Caron, Jean-François. *The Moral Dilemmas of Fighting Terrorism and Guerrilla Groups*. 1st ed. Vol. 2 (Germany: De Gruyter, 2023). https://doi.org/10.1515/9783110757569.

51 Harmer, Tanya. *Allende’s Chile and the Inter-American Cold War*. New edition 1. (Chapel Hill: The University of North Carolina Press, 2011) https://doi.org/10.5149/9780807869246. John Dinges. *The Condor Years: How Pinochet and His Allies Brought Terrorism to Three Continents* (New York: The New Press, 2012); Naomi Roht-Arriaza. *The Pinochet Effect*, (Philadelphia, US: University of Pennsylvania Press, Inc, 2011)’ Tanya Harmer. ‘Fractious Allies: Chile, the United States, and the Cold War, 1973–76’. *Diplomatic History* 37, no. 1 (2013): 109–43. https://doi.org/10.1093/dh/dhs005

52 Waitzkin, Howard, Perez, Alina, Anderson, Matthew. *Social medicine and the coming transformation* (New York, New York: Routledge, 2021); Howard Waitzkin, ‘Medicine and Social Change: Lessons from Chile and Cuba’, Chap. 7 in *The Second Sickness: Contradictions of Capitalist Health Care,* 2nd ed. (Lanham, MD: Rowman & Littlefeld, 2000); and Illane, María Angélica, *‘En el Nombre del Pueblo, del Estado y de la Ciencia--’: Historia Social de la Salud Pública, Chile 1880–1973* (Santiago de Chile: Colectivo de Atención Primaria, 1993).

53 Howard Waitzkin, ‘Social Medicine at home and abroad’. In Anne-Emanuelle Birn and Theodore M. Brown. *Comrades in Health : U.S. Health Internationalists, Abroad and at Home* (New Brunswick, NJ: Rutgers University Press, 2013): 153; Waitzkin, Howard. ‘Health and Praxis: Social Medicine in Latin America’. *Medicine and Public Health at the End of Empire* (Boulder, Colo.; Paradigm Publishers, 2011): 156; Howard Waitzkin. ‘Social Medicine in Latin America’. In Howard Waitzkin, Alina Perez, Matthew Anderson. *Social Medicine and the Coming Transformation* (New York, NY: Routledge, 2020): 177

54 Salvador Allende. *La Realidad Medico-Social Chilena* (Santiago de Chile: Ministerio de Salubridad, Prevencion y Asistencia Social, 1939): 6–8.

55 Waitzkin ‘Social Medicine’ In Waitzkin et al *Social Medicine,* (New York, 2020):179.

56 Edwards, Sebastian. *The Chile Project: The Story of the Chicago Boys and the Downfall of Neoliberalism*. First edition (Prince­ton, New Jersey: Prince­ton University Press, 2023). https://doi.org/10.1515/9780691249360; Clark, Timothy David. ‘Rethinking Chile’s ‘Chicago Boys’: Neoliberal Technocrats or Revolutionary Vanguard?’ *Third World Quarterly* 38, no. 6 (2017): 1350–65. https://doi.org/10.1080/01436597.2016.1268906; Taylor, Marcus. *From Pinochet to the ‘Third Way’: Neoliberalism and Social Transformation in Chile* (London; Pluto Press, 2006); Araujo, Kathya. *The Circuit of Detachment in Chile: Understanding the Fate of a Neoliberal Laboratory*. 1st ed. (Cambridge, United Kingdom: Cambridge University Press, 2022).

57 Founded in the mid-1960s, the *MIR* was radical Marxist-Leninist collective aggregating student activist, syndicalist, and former members of traditional leftist parties in Chile. At its core, the MIR broke away from the passive role communist and socialist parties played during the earlier crisis of living in Chile and advocated for all forms of armed resistance *a la Guevarismo.* Though this organization was not part of Allende’s *Unidad Popular UP* movement, it explicitly supported the democratic process it led, becoming a prime target in Pinochet’s genocide of the left. For details, see Stone, Nathan J. ‘Prevention or Provocation? How U.S. Support for Christian Democrats Radicalized Chile’s MIR, 1964–1970’. *The Latin Americanist* 65, no. 1 (2021): 142–68. https://doi.org/10.1353/tla.2021.0008; Téllez Lúgaro, Eduardo, Felipe Novoa Saldías, and Nicolás Iturra Fonseca. ‘El Movimiento de Izquierda Revolucionaria (MIR) de Chile según la CIA, 1967–1971’. *Cuadernos de historia (Santiago, Chile)*, no. 58 (2023): 333–53. https://doi.org/10.5354/0719-1243.2023.71047; Ruiz, María Olga. ‘Disciplina y Desacato: Mandatos Militantes y Traición En El Movimiento de Izquierda Revolucionaria (MIR) En Chile’. *Nuevo Mundo, Mundos Nuevos*, 2013. https://doi.org/10.4000/nuevomundo.64899.

58 Archivo Nacional de Chile. ‘Testimonio de Maria Isabel Matamala, exmilitante del MIR’. Available at: https://youtu.be/xUgHjuL6rv4?si=8s1COb8Nvdttz8JV (accessed on 2 March, 2025).

59 Villa Grimaldo. ‘Entrevista a Maria Isabel Matamala’. Available at: https://www.youtube.com/live/gFM5swdtIM0?si=EVIOGrF3bhxPNyXk (accessed on 2 March, 2025).

60 Cruz, Enrique, Marilyn Mulford, Quique Cruz, production company Interfaze Educational Productions, *Archeology of Memory: Villa Grimaldi* (Berkeley, CA: Berkeley Media, 2008); Frenz, Helmut. ‘A Bishop Saving “Singers”: Tales of Torture in Pinochet’s Chile’. *Dialog : A Journal of Theology* 47, 3 (2008): 251–60. https://doi.org/10.1111/j.1540-6385.2008.00399.x; Meade, Teresa. ‘Holding the Junta Accountable: Chile’s “Sitios de Memoria” and the History of Torture, Disappearance, and Death’. *Radical History Review* 79 (2001): 123–39. https://doi.org/10.1215/01636545-2001-79-123.

61 Howard Waitzkin and Hilary Modell, ‘Medicine, Socialism, and Totalitarianism: Lessons from Chile’. *New England Journal of Medicine 291* (1974):171–177.

62 For a comprehensive review of the Argentinian dictatorship, see Julieta Carla Rostica, ‘Apuntes ‘Triple A’. Argentina, 1973–1976’, *Desafíos* 23, no. 2 (December 16, 2011): 21–52, https://www.redalyc.org/pdf/3596/359633170007.pdf; Heloisa Cristina Ribeiro, ‘A Ditadura Militar Na Argentina (1976–1983): O Aparato Repressivo e a Justiça de Transição’ 10 (April 14, 2021): 100–115, https://doi.org/10.11606/ISSN.1982-7547.HD.2021.159255; Yañez, César Manuel Román. ‘Crisis, State of Emergency and State Terrorism: Particularities of the Dictatorship in Argentina (1976−1983)’. *Iberoamerikanskie Tetradi* 10, no. 3 (2023): 55–69. https://doi.org/10.46272/2409-3416-2022-10-3-55-69.

63 COSPA was founded in Mexico in response to the brutal Argentinian military coup, becoming a key node in the international network of resistance. The collective was central to the human rights activism of the time, playing a crucial role in denouncing state terror and supporting its victims. For details, see Graciela Graschinsky de Cohan, ‘Histoire, Migration et Déracinement : Le Legs de Marie Langer’, *Topique* 80, no. 3 (September 1, 2002): 63–79, https://doi.org/10.3917/TOP.080.0063; Pablo Yankelevich, ‘The COSPA: A Political Experience of the Argentine Exile in Mexico’, *Latin American Perspectives* 34, no. 4 (July 1, 2007): 68–80, https://doi.org/10.1177/0094582X07302947; José María Casco, ‘The Intelectual Exile in Mexico. Notes on the Argentinean Experience 1974–1983’, *Apuntes de Investigación Del CECYP*, no. 13 (January 1, 2008): 149–64, https://dialnet.unirioja.es/servlet/articulo?codigo=4509239.

64 The 1979 Sandinista Revolution in Nicaragua was a watershed moment in Latin American history, inspiring a wave of international solidarity from activists, intellectuals, and institutions sympathetic to its goals of social justice and national sovereignty. Within this global movement, Mexico's UAM-X held a unique position, becoming a key partner in the Sandinista project of social reconstruction, which included the establishment of a healthcare system. Details on the collaborations, see Richard Garfield and Williams, Glen. *Healthcare in Nicaragua: Primary care under changing regimes* (New York, US: Oxford University Press, 1992); Lira, Elizabeth, and Eugenia Weinstein. *Psicoterapia y represión política* (Mexico City, Mexico: Siglo XXI Editores, 1984); Diaz-Briquets, Sergio, and John M Donahue. ‘The Nicaraguan Revolution in Health: From Somoza to the Sandinistas’. *The Hispanic American Historical Review* 67, no. 2 (1987): 346. https://doi.org/10.2307/2515041.

65 The UAM-X's innovative modular system (‘sistema modular’) eliminated traditional academic departments and instead organized learning around ‘problem-objects’ derived from social reality. This model, combined with an explicit political mandate for social engagement (‘vinculación’), cultivated a generation of professionals trained to work within community settings and critically analyse social issues. This pedagogical philosophy, which aimed for critical and socially oriented approaches, resonated deeply with Stolkiner’s background in Plataforma/CID psy-activism and became a central focus of her psy-methods. Details in De Ibarrola, María. *Las dimensiones sociales de la educación superior* (Mexico City, Mexico: SEP/Consejo Nacional de Fomento Educativo, 1985); Arbesú, Isabel y Luis Berruecos. *El Sistema Modular en la Universidad Autónoma Metropolitana* (México City, Mexico: UAM-X, 1996); Manjares et al., *Bases Conceptuales y Sistema Modular: Una reflexión colectiva* (Mexico City, Mexico: UAM-X/COPLADA, 2005).

66 Interview with Alicia Stolkiner, October 2018. Also document this second dictatorship.

67 The *Plataforma* within APA emerged inspired by the late 1960s European upheaval in the field. In 1969, the International Psychoanalytical Association Conference in Rome witnessed the de-branching of a subsector of the psychoanalysis collective for political and ideological reasons. This was the birth of the ‘Platform Movement’, later expanding to Austria, Italy and Switzerland, and which happened to be integrated by various Argentinian professionals who took the anti-imperialist/anti-capitalist experience back to the country. The movement then articulated with emerging nationalist perspectives who viewed their identities as non-aligned, postcolonial stances away from the structural dependency to US-USSR superpowers. The new *Plataforma* grew as part of the largest psychiatric association in Argentina, the *Federacion Argentina de Psiquiatria* FAP, that integrated Marxist thinking with anti-colonial authors such as Fanon, Freire, ‘Che’ Guevara, and Mao Zedong. For details, see Nick Totton, *The Politics of Psychotherapy: New Perspectives* (US: Open University, 2006)*;* Enrique Carpintero, Alejandro Vainer, *Huellas de la Memoria* (Buenos Aires: Editorial Topia, 2005); McMahon, Robert J. *The Cold War in the Third World*. 1st ed. Oxford: Oxford University Press, Incorporated, 2013); Bosteels, Bruno. ‘Critique of Planned Obsolescence: Marx and Freud in Latin America’. *Revista Hispánica Moderna* 64, no. 1 (2011): 23–37. https://doi.org/10.1353/rhm.2011.0000.

68 Multiple psy-collectives were involved in the resistance against the emerging regimen in times of dictatorship, as the institutionalization of service became a fertile ground for political contestations – both to reinforce authoritarianism and to propose alternatives. Details in Marco A. Ramos, ‘Psychiatry, Authoritarianism, and Revolution: The Politics of Mental Illness during Military Dictatorships in Argentina, 1966–1983’, *Bulletin of the History of Medicine* 87, no. 2 (January 1, 2013): 250–78, https://doi.org/10.1353/BHM.2013.0029; Plotkin, Mariano Ben. *Freud in the Pampas: The Emergence and Development of a Psychoanalytic Culture in Argentina*. 1st ed. (Stanford, Calif: Stanford University Press, 2001); Rabbia, Hugo H, Silvina Brussino, and Débora Imhoff. ‘Psicología Política en Argentina: un recorrido por la historia de una disciplina emergente’. *Revista psicologia política* 10, no. 20 (2010): 199–213.

69 Stolkiner was appointed staff shortly after graduation and kept a close affiliation to Plataforma and the CID. For details, see Braunstein, Nestor, Pasternac, Marcelo, Benedito, Gloria, Saal. *Psicologia: Ideologia y Ciencia.* (Cerro del Agua, Mexico: Siglo Veintiuno Editores, 1975 – 8th edition, 1982).

70 The persecution of the psy-disciplines was not an incidental byproduct of the repression, but a strategic objective of the dictatorship. Argentina had developed a widespread ‘psychoanalytic culture’, unparalleled in the world, where Freudian and Lacanian ideas were not confined to the clinic but were central to university curricula, social sciences, and a middle-class public sphere that was increasingly critical and politicized. This intellectual effervescence, particularly its linkage with leftist politics and social critique in the 1960s and early 1970s, made the entire field a target. The dictatorship's ideology was based on a paranoid vision of a healthy, hierarchical, Catholic ‘social body’ infected by the ‘disease’ of subversion. Psychoanalysis and critical psychology, with their questioning of authority, exploration of sexuality, and focus on non-conformity, were seen as primary vectors of this ideological contagion. For details on the repression, see Carpintero, Enrique, and Alejandro Vainer. 2006. *Las huellas de la memoria: Psicoanálisis y salud mental en la Argentina de los '60 y '70 – Tomo II (1970–1983).* 2nd Ed. (Buenos Aires, Argentina: Topia, 2018); Hollander, N C. ‘Psychoanalysis confronts the politics of repression: the case of Argentina’. *Social science & medicine* 28, 7 (1989): 751–8. doi:10.1016/0277–9536(89)90223–2; Vezzetti, Hugo. *Aventuras de Freud en el país de los argentinos: De José Ingenieros a Enrique Pichon-Rivière* (Buenos Aires, Argentina: Paidós, 1996).

71 Interview with Alicia Stolkiner, July 2021.

72 For details on the case, see Giraudo, Marina, and Laura Bonafé. ‘Las Intervenciones Económicas Estatales Sobre El Grupo Mackentor Durante El Terrorismo de Estado’. *H-Industri@ (Ciudad Autónoma Buenos Aires)* 17, no. 33 (2023): 99–116. https://doi.org/10.56503/H-Industria/n.33(17)pp.99-116; Vega, Juan Carlos. *Caso Mackentor: el valor de la condena de la CIDH a la Argentina por violar los derechos humanos*. *CE Noticias Financieras*. Spanish ed. Miami: ContentEngine LLC, a Florida limited liability company, 2022; *Mackentor, sin resarcimiento a 37 años de la apropiacion: Source: NoticiasFinancieras*. *NoticiasFinancieras* (Miami: Global Network Content Services LLC, DBA Noticias Financieras LLC, 2014).

73 Interview with Alicia Stolkiner, July 2021.

74 To clarify, the origin of the social medicine framework, based on Marx’s historical materialism, remains a subject of debate. The general agreement highlights a range of actors and influences, with Jaime Breilh and Asa Cristina Laurell playing pivotal roles. Nevertheless, it is clear that a network of supporters and scholarly backing contributed to the consolidation and expansion of the model, including Stolkiner and the critical psychology network. This is particularly relevant as the UAM-X adapted its approach in response to the fall of the USSR, the rise of neoliberalism, and the turn of the century (globalization and expansion of free trade). For details, see Granados Cosme, José Arturo, and Guadalupe Delgado Sánchez. ‘La Medicina Social en la Universidad Autónoma Metropolitana: Análisis de tesis de la Maestría en Medicina Social’. *Salud Problema/*Nueva Época, no. 18–19 (2005): 43–54; Mercado Martínez, Francisco Javier, and Leticia Robles Silva. ‘La Medicina Social Latinoamericana y sus miradas múltiples sobre la investigación cualitativa’. *Salud Problema*/Nueva Época, no. 17 (2004): 39–45; Fonseca, Sebastian. ‘A “Counter-Hegemonic” Social Medicine: Leftist Physicians during the Latin American Cold War’. Chapter In *Medicine on a Larger Scale: Global Histories of Social Medicine*, edited by Anne Kveim Lie, Jeremy A. Greene, and Warwick Anderson (Cambridge: Cambridge University Press, 2025): 167–85.

75 On the social determination model, see Castro, Roberto. ‘Medical Sociology in Latin America’. In *The Oxford Handbook of the Sociology of Latin America*, edited by Liliana Rivera-Sánchez and Xóchitl Bada (Oxford University Press, 2020): 572–88. https://doi.org/10.1093/oxfordhb/9780190926557.013.35; Samaja, Juan. *Epistemología de la salud: reproducción social, subjetividad y transdisciplina* (Buenos Aires, Argentina: Lugar Editorial, 2004); Michael Harvey, Carlos Piñones Rivera, and Seth M. Holmes, ‘Thinking with and Against the Social Determinants of Health: The Latin American Social Medicine (Collective Health) Critique from Jaime Breilh’, *International Journal of Health Services* 52, no. 4 (September 1, 2022): 433–41, https://doi.org/10.1177/00207314221122657; Elis Borde, Mario Hernández-Álvarez, and Marcelo Firpo de Souza Porto, ‘Uma Análise Crítica Da Abordagem Dos Determinantes Sociais Da Saúde a Partir Da Medicina Social e Saúde Coletiva Latino-Americana’ *Saúde debate* 39, no. 106 (2015): 841–54, https://doi.org/10.1590/0103-1104201510600030023.

76 Collective mental health represents a radical theoretical, clinical, and political project that offers a fundamental critique of traditional, individualizing, and often pathologizing models of psychiatry and psychology. Its core assertion is that collective forms of mental distress are not primarily rooted in individual biology or psyche but are actively produced by oppressive social, political, and economic structures. For more, see Capella, Manuel. ‘Salud Mental Colectiva y Determinación Social: Posibilidades Paradigmáticas’. *Cuadernos de psicología (Bellaterra, Barcelona)* 25, no. 2 (2023): e1935. https://doi.org/10.5565/rev/qpsicologia.1935; Hernández-Holguín, Dora María, Beatriz Elena Arias López, and Ángel Martínez-Hernáez. ‘Collective Mental Health: A Review of the Concept in the Academic Literature of Brazil, Colombia, and Spain’. *Saúde e Sociedade* 32, no. 3 (2023). https://doi.org/10.1590/s0104-12902023210693en; Stolkiner, Alicia. ‘La Subjetividad en la Salud: de la Producción Social a la Práctica Institucional’. In *La Producción Social de la Subjetividad*, edited by Alicia Stolkiner et al. (Buenos Aires, Argentina: Lugar Editorial, 1999); Stolkiner, Alicia. ‘Conceptualizando la salud mental en las prácticas: consideraciones desde el pensamiento de la medicina social/salud colectiva latinoamericanas’. *Vertex: Revista Argentina de Psiquiatría* 24, no. 111 (2013): 219–26.

77 Susana Belmartino ‘Trayectoria personal y colectiva en la construcción de la historia de la Salud Publica’ Interview by Galeano, D. (Buenos Aires: CeDoPS; 2010). Available at: https://youtu.be/Xrn0B3b5znE?si=f4y3NDKR9IPgbF2J (accessed on 20 December, 2024)

78 A key protagonist in the Argentinian state-sponsored terror was the ‘Concentración Nacionalista Universitaria’ (CNU), a far-right university group that evolved from a campus-based shock troop into a professionalized paramilitary death squad. While the CNU operated in several cities, its branch in the university city of La Plata was particularly notorious for its brutality, acting as a foundational component of the ‘Alianza Anticomunista Argentina’ (Triple A) and a direct precursor to the methodology of the subsequent dictatorship. Details in Ladeuix, Juan Iván. ‘Sindicatos, Partido, Universidad y Poder Judicial: El papel de las derechas peronistas en la configuración de un complejo contrainsurgente. Mar del Plata 1970–1976’. *Cuadernos de Marte: revista latinoamericana de sociología de la guerra*, no. 21 (2021): 97–128; Daniel Cecchini and Alberto Elizalde Leal. *La CNU: El terrorismo de Estado antes del golpe* (Buenos Aires, Argentina: Una investigación de Miradas al Sur, 2013); Lvovich, Daniel. *El nacionalismo de derecha: Desde sus orígenes a Tacuara* (Buenos Aires, Argentina: Capital Intelectual, 2006).

79 Menendez, E. ‘Entrevista a Eduardo Menendez’. Interview completed for the *XXXI Congreso FAMG 2016 (XXXI Argentinian General Medicine Federation).* Available at: https://youtu.be/VahPQFFq-YQ (accessed on 15 January, 2025).

80 Ibid.

81 Details in Spinelli, H., Librandi, J. M., & Zabala, J. P. ‘Cuadernos médico sociales from Rosario, Argentina, and other Latin American social medicine journals from the 1970s and 1980s’. *História, Ciências, Saúde - Manguinhos, 24, no.* 4 (2017), 877–895. doi:https://doi.org/10.1590/s0104-59702017000500002.

82 Ibid. Cebes stands for the *Centro Brasileiro de Estudos de Saude* Brasilian (Centre for the Studies of Health) – an independent research center founded in 1976 as the basis for the *Sanitarismo* movement. For details on the institution, see Reis, Tiago Siqueira. ‘The Brazilian Center for Health Studies and the Brazilian Association of Collective Health: The Issue of the Nationalization of Health in the Brazilian Public Health Reform, 1976–1988’. *História, Ciências, Saúde–Manguinhos* 31 (2024): e2024030-. https://doi.org/10.1590/S0104-59702024000100030en; Jacobina, André Teixeira. ‘A Relação Do Cebes Com o PCB Na Emergência Do Movimento Sanitário’. *Saúde Em Debate (Londrina, Brazil)* 40, no. spe (2016): 148–62. https://doi.org/10.1590/0103-11042016s13; Rizzotto, Maria Lucia Frizon, Ana Maria Costa, and Lenaura de Vasconcelos Costa Lobato. ‘Justiça Social, Democracia Com Direitos Sociais e Saúde: A Luta Do Cebes’. *Saúde Em Debate* 42, no. 116 (2018): 5–10. https://doi.org/10.1590/0103-1104201811600.

83 Collaborative publications between scholars from Argentina and Brazil testify to the close relationships. For instance, a solid tie was built between Belmartino and Fleury – which developed into publications like *Reshaping Health Care in Latin America: A Comparative Analysis of Health Care Reforms in Argentina, Brazil, and Mexico* (Ottawa, ON: International Development Research Centre, 2000).

84 Details on the methods in Tarrés, María Luisa. ‘Observar, escuchar y comprender. Sobre la tradición cualitativa en la investigación social en México’. *Revista del Centro de Investigación de la Universidad la Salle* 10, no. 37 (2012): 113–26; Jan C. Behrends and Thomas Lindenberger eds. *Underground Publishing and the Public Sphere: Transnational Perspectives* (Wiener: LIT Verlag, 2014), http://ci.nii.ac.jp/ncid/BB18892935.

85 Expanded analysis on such methods of diffusion in the Latin American Cold War, see Escorel, Sarah. 2008. ‘História da Reforma Sanitária e do SUS: um percurso e um balanço’. *Ciência & Saúde Coletiva* 13 (2): 23–45; Lucy Bell and Patrick O’Hare, ‘Latin American Politics Underground: Networks, Rhizomes and Resistance in Cartonera Publishing’, *International Journal of Cultural Studies* 23, no. 1 (January 1, 2020): 20–41, https://doi.org/10.1177/1367877919880331; María Josefina Lamaisón, ‘Editar a Partir Do Exílio: Os Informativos CELATS-ALAETS Como Exercício de Resistência (1976–1985)’ 21, no. 30 (July 19, 2021): 76–103, https://doi.org/10.46752/ANPHLAC.30.2021.3975.

86 Kelly, Patrick William. *Sovereign Emergencies: Latin America and the Making of Global Human Rights Politics* (Cambridge: Cambridge University Press, 2018). Samantha Viz Quadrat, ‘A emêrgencia do tema dos direitos humanos na América Latina’, in Carlos Fico, Marieta de Moraes Ferreira, Maria Paula Araujo, and Samantha Viz Quadrat, eds., *Ditadura e democracia na América Latina: balanço histórico e perspectivas* (Rio de Janeiro: Fundação Getúlio Vargas, 2008), 361–95; Jay Winter, *Dreams of Peace and Freedom: Utopian Moments in the Twentieth Century* (New Haven, CT: Yale University Press, 2008).

87 Hatzky, Christine, and Jessica Stites Mor. ‘Latin American Transnational Solidarities: Contexts and Critical Research Paradigms’. *Journal of Iberian and Latin American Research* 20, no. 2 (2014): 127–40. https://doi.org/10.1080/13260219.2014.939121; Pries, Ludger, and Pablo Yankelevich. ‘Comparing Contexts, Institutions and Periods of the Émigrés’ Arrival and Possible Return’. In *European and Latin American Social Scientists As Refugees, Émigrés and Return-Migrants*, 285–95 (Switzerland: Springer International Publishing AG, 2018). https://doi.org/10.1007/978-3-319-99265-5_13; Paul Durrenberger, E. ‘Solidarity: Latin America and the United States Left in the Era of Human Rights’. *Anthropology of Work Review* 41, no. 1 (2020): 61–62. https://doi.org/10.1111/awr.12184.

88 Helmut Frenz, *Mi vida chilena: Solidaridad con los oprimidos* (Santiago: LOM, 2006); Allan R. Brewer-Carías, *Constitutional Protection of Human Rights in Latin America: A Comparative Study of Amparo Proceedings* (New York, NY: Cambridge University Press, 2009); Margaret Power, ‘The U.S. Movement in Solidarity with Chile in the 1970s’, *Latin American Perspectives* 36, no. 6 (2009): 46–66.

89 Stites Mor, Jessica. *Human Rights and Transnational Solidarity in Cold War Latin America*. 1st ed. (Madison: University of Wisconsin Press, 2013): 8.

90 Tobias Kelly, *This Side of Silence: Human Rights, Torture, and the Recognition of Cruelty* (Philadelphia, PA: University of Pennsylvania Press, 2012); Tobias Kelly, ‘What We Talk About When We Talk About Torture’, *Humanity*, 2, 2 (2011): 327–43; Samuel Moyn, ‘From Antiwar Politics to Antitorture Politics’, in Austin Sarat, Lawrence Douglas, and Martha Umphrey, eds., *Law and War* (Palo Alto, CA: Stanford University Press, 2014): 154–97; Harmer, Tanya. ‘The View from Havana: Chilean Exiles in Cuba and Early Resistance to Chile’s Dictatorship, 1973–1977’. *The Hispanic American Historical Review* 96, no. 1 (2016): 109–46. https://doi.org/10.1215/00182168-3423904; Jadwiga Pieper-Mooney, ‘Chilean Exile in the German Democratic Republic (GDR): The Politics of Solidarity in the Cold War’, in Kim Christiaens, Idesbald Goddeeris, and Magaly Rodríguez Garcia, eds., *European Solidarity with Chile* (Berlin: Peter Lang, 2013): 275–99.

91 Birn and Muntaner. ‘Latin American Social Medicine across Borders’ *Global Public Health* 14, no. 6–7 (2019): 817–34; Borde and Hernández. ‘Revisiting the social determinants of health’ *Global Public Health* 14, no. 6–7 (2019): 847; Carrillo Roa A, Santana JP. ‘Regional integration and South-South cooperation in health in Latin America and the Caribbean’. *Rev Panam Salud Pública 32 (2012):368–75;* Riggirozzi P. ‘Regionalism through social policy: collective action and health diplomacy in South America’. *Econ Soc 43 (2014):432–54.*

92 Carter, *In Pursuit of Health Equity*; Breilh, *Critical Epidemiology*; Franco et al., *Debates*.

93 Jeremi Suri, *Power and Protest: Global Revolution and the Rise of Détente* (Cambridge, MA: Harvard University Press, 2003); Gould, ‘Solidarity under Siege’ *American Historical Review*, vol. 114, no. 2 (April 2009), 348–75; O’Sullivan, Kevin. ‘Civil War in El Salvador and the Origins of Rights-Based Humanitarianism’. *Journal of Global History* 16, no. 2 (2021): 246–65. https://doi.org/10.1017/S1740022820000170.

94 Silvina Jensen, *Los exiliados: la lucha por los derechos humanos durante la dictadura* (Buenos Aires: Sudamericana, 2010); Katherine Hite, *When the Romance Ended: Leaders of the Chilean Left, 1968–1998* (New York, NY: Columbia University Press, 2000); Mario Sznajder and Luis Roniger, *The Politics of Exile in Latin America* (New York, NY: Cambridge University Press, 2009).

95 This movement was supported internationally by the likes of the World Council of Churches, the Vatican, and the Catholic University of Louvain in Belgium, and integrated protestant church and Jewish collectives. For details, see Phillip Berryman, *Liberation Theology: Essential Facts About the Revolutionary Movement in Latin America and Beyond* (Philadelphia, PA: Temple University Press, 1998); Serbin, Ken. *Secret Dialogues: Church-State Relations, Torture, and Social Justice in Authoritarian Brazil* (Pittsburgh, US: University of Pittsburgh Press, 2000); Charles Harper, *O Acompanhamento: Ecumenical Action for Human Rights in Latin America, 1970–1990* (Geneva: WCC Publishing, 2006).

96 Daniel H. Levine, ‘Assessing the Impacts of Liberation Theology in Latin America’, *The Review of Politics*, 50, 2 (1988): 241–63. On Brazil, see John Burdick, *Looking for God in Brazil: The Progressive Catholic Church in Urban Brazil’s Religious Area* (Berkeley, CA: University of California Press, 1996); Robin Nagle, *Claiming the Virgin: The Broken Promise of Liberation Theology in Brazil* (New York, NY: Routledge, 1997); Mark Engler, ‘Toward the “Rights of the Poor”: Human Rights in Liberation Theology’, *Journal of Religious Ethics*, vol. 28, no. 3 (Fall 2000), 339–65.

97 Kenneth Serbin, *Secret Dialogues: Church-State Relations, Torture, and Social Justice in Authoritarian Brazil* (Pittsburg, PA: University of Pittsburg Press, 2000); Charles R. Harper, *Ecumenical Action for Human Rights in Latin America, 1970–1990* (Geneva: World Council of Churches, 2006); Paulo César Gomes, *Os bispos católicos e a ditadura militar brasileira: a visão da espionagem* (Rio de Janeiro: Record, 2014).

98 Critical perspectives on the state of global health today can be found in Andrew Lakoff, ‘Two Regimes of Global Health’, *Humanity* 1, no. 1 (January 1, 2010): 59–79, https://doi.org/10.1353/HUM.2010.0001; João Biehl and Adriana Petryna, ‘Peopling Global Health’, *Saude E Sociedade* 23, no. 2 (June 1, 2014): 376–89, https://doi.org/10.1590/S0104-12902014000200003; Jeremy Youde, ‘The Rockefeller and Gates Foundations in Global Health Governance’, *Global Society* 27, no. 2 (March 14, 2013): 139–58, https://doi.org/10.1080/13600826.2012.762341.

99 Thaiane Oliveira and Tatiane Mendes Pinto, ‘Knowledge and Emancipation’, *Journal of Digital Social Research* 6, no. 3 (November 1, 2024): 40–58, https://doi.org/10.33621/jdsr.v6i3.33244; Heping Xie, ‘Making Disappearance Visible’, *The American Historical Review* 127, no. 2 (June 1, 2022): 664–90, https://doi.org/10.1093/ahr/rhac153; Ramin Asgary, ‘Direct Killing of Patients in Humanitarian Situations and Armed Conflicts: The Profession of Medicine Is Losing Its Meaning’, *American Journal of Tropical Medicine and Hygiene* 92, no. 4 (April 1, 2015): 678–80, https://doi.org/10.4269/AJTMH.14-0364

